# Transcription Factors Active in the Anterior Blastema of *Schmidtea mediterranea*

**DOI:** 10.3390/biom11121782

**Published:** 2021-11-28

**Authors:** Yoko Suzuki-Horiuchi, Henning Schmitz, Carlotta Barlassina, David Eccles, Martina Sinn, Claudia Ortmeier, Sören Moritz, Luca Gentile

**Affiliations:** 1 Planarian Stem Cell Laboratory, Max Planck Institute for Molecular Biomedicine, Von Esmarch-Str. 54, 48149 Münster, Germany; yokoho@pennmedicine.upenn.edu (Y.S.-H.); henning.schmitz@outlook.com (H.S.); carlotta.barlassina@gmail.com (C.B.); ortmeier@mpi-muenster.mpg.de (C.O.); soeren.moritz@uk-koeln.de (S.M.); 2 Max Planck Institute for Molecular Biomedicine, Röntgenstr. 20, 48149 Münster, Germany; bioinformatics@gringene.org (D.E.); martina.sinn@mpi-muenster.mpg.de (M.S.); 3 Pluripotency & Regeneration Laboratory, Department Animal Physiology, University of Osnabrück, Barbarastr. 11, 49076 Osnabrück, Germany

**Keywords:** stem cells, transcription factors, regeneration, pluripotency, differentiation, planarian, blastema, RNA-seq

## Abstract

Regeneration, the restoration of body parts after injury, is quite widespread in the animal kingdom. Species from virtually all Phyla possess regenerative abilities. Human beings, however, are poor regenerators. Yet, the progress of knowledge and technology in the fields of bioengineering, stem cells, and regenerative biology have fostered major advancements in regenerative medical treatments, which aim to regenerate tissues and organs and restore function. Human induced pluripotent stem cells can differentiate into any cell type of the body; however, the structural and cellular complexity of the human tissues, together with the inability of our adult body to control pluripotency, require a better mechanistic understanding. Planarians, with their capacity to regenerate lost body parts thanks to the presence of adult pluripotent stem cells could help providing such an understanding. In this paper, we used a top-down approach to shortlist blastema transcription factors (TFs) active during anterior regeneration. We found 44 TFs—31 of which are novel in planarian—that are expressed in the regenerating blastema. We analyzed the function of half of them and found that they play a role in the regeneration of anterior structures, like the anterior organizer, the positional instruction muscle cells, the brain, the photoreceptor, the intestine. Our findings revealed a glimpse of the complexity of the transcriptional network governing anterior regeneration in planarians, confirming that this animal model is the perfect playground to study in vivo how pluripotency copes with adulthood.

## 1. Introduction

Planarians can regenerate all the missing tissues and organs after amputation, like the central nervous system (CNS) [1,2,3,4,5,6,7,8,9,10], the photoreceptors [11,12,13,14,15,16,17,18,19,20,21], the protonephridia [22,23,24], the intestine [25,26,27,28], and the body wall [29,30,31]. Planarian regeneration is a complex process where the missing tissues are both rebuilt and properly integrated with the existing tissues. It hinges on the presence of neoblasts, which are a heterogeneous population of adult stem cells among which both uncommitted pluripotent and committed multipotent cells linger [32,33,34,35,36,37,38]. Many genes, especially transcriptional regulators, were identified that are involved in planarian regeneration, either regulating its temporal and spatial organization, like *MyoD* [29], *Prep* [39], *Isl-1* [40], or contributing directly to the cell and tissue functional differentiation, from the exit from pluripotency (e.g., *Tcf15*) to the terminal differentiation of specific tissues, like the brain, the intestine, the photoreceptors, the protonephridia and the germline [8,14,22,25,41,42,43,44,45]. Also, a number of papers has been published based on the generation of planarian transcriptomes since 2010, detailed in Table 1 [3,14,28,34,36,46,47,48,49,50,51,52,53,54,55,56,57,58,59,60,61,62,63,64,65,66,67,68]. These works coupled RNA-Seq, bioinformatic and functional RNAi analyses in order to depict and dissect the complexity of the transcriptional landscape of planarian regeneration [3,14,28,52,55,56,57,66,67,68,69,70,71,72]. However, what is not yet fully understood is how the blastema is temporally and spatially regulated as a whole, and how the regeneration of several structures of the body is concerted.

A previous effort aimed to discover key regulators of regenerations differentially expressed between head and tail was made [57] which was based on the collection and sequencing of the entire regenerating fragments. Therefore, we focused on a top-down approach where, alongside with regenerating anterior blastema samples, we also included stem-cell-enriched (e.g., X1 cell population), progeny-enriched (e.g., X2 cell population) stem-cell-depleted (Xin cell population, *SmB*(RNAi) planarians) and stem cell- and progeny-depleted (e.g., irradiated planarians) samples. By applying an *ad-hoc* bioinformatic pipeline to our RNA-Seq dataset, we shortlisted several transcription factors (TFs) that take part in the *bona fide* regulatory network of genes controlling adult pluripotent stem cells (aPSCs) commitment and differentiation during anterior regeneration, and functionally validated them by means of RNAi in regenerating planarians.

To our knowledge, this is the first attempt to describe the anterior regeneration in planarian from the transcriptional regulatory point, comparing the dissected blastema with other non-regenerating tissues. Further works are needed to depict the whole complexity of this network and to understand how it mechanistically concerts the differentiation of the SCs into terminally differentiated cells, to achieve complete regeneration. Thanks to the opportunity of studying pluripotency in vivo in the adult, planarian is a reference model system where pluripotency-based regeneration hypotheses could be tested, applicable for the future treatment of human diseases.

## 2. Materials and Methods

### 2.1. Planarian Husbandry

The asexual strain of *S. mediterranea* (clonal line BCN10) were kept in 1× planarian artificial medium (PAM) at 20 °C with 12 h shift of the light/dark cycle, and fed twice a week with organic bovine liver. The animals used for the experiments were starved for at least one week prior to the experiments. To obtain head, trunk, or tail fragments, intact animals were amputated by a scalpel blade pre- and post-pharyngeally, respectively.

### 2.2. Preparation of Blastema Samples

Regenerating head fragments were dissected at 3 or 6 days post amputation (dpa). For dissection, animals were placed on a glass slide and excess water was removed. The glass slides were placed on crushed ice under the microscope. Blastema were dissected with a new scalpel blade and collected in Trizol^®^. After the blastema was removed, a stripe of tissue approx. 0.5–1 mm wide (postblastema) was also removed and stored in Trizol^®^. Eventually, the non-regenerating part of the fragment (rest of body, RoB) was also collected in Trizol.

### 2.3. Flow Cytometry Analysis and FACS

To collect X1, X2, Xin cells, homeostatic animals were treated with 2% L- Cysteine in CMFH (pH 7.0) for 2 min in 100 mm petri dish placed on ice, rinsed in CMFH, transferred to a cover-slip and cut into several pieces using a scalpel blade no. 29. The fragments were transferred into a 1.5 mL protein low-binding tube (Protein LoBind, Eppendorf, Hamburg, Germany) using 250 μL of CMFH. For the cell dissociation, 250 μL 2× papain-solution was added and the fragments were incubated for 60 min at 25 °C. The reaction was stopped with 500 μL of 2× stop solution and the digested tissue was passed about 20 times in a large borehole P1000 pipette (STARLAB 1000 μL XL). The suspension was passed through a 30 µm filter (CellTrics, Parted, Görlitz, Germany) and spun down for 5 min at 500× *g* at 4 °C. After resuspension in 1 mL CMFH the cells were counted and the concentration was adjusted to 5 × 105 cells/mL. After adding Hoechst and Calcein to a final concentration of 10 μg/mL and 0.05 μg/mL, respectively, the cell suspension was incubated for 2 h at RT on a horizontal shaker in the dark. The suspension was again filtered, centrifuged for 5 min at 500× *g* at 4 °C and resuspended in 500 μL CMFH. After addition of 1 μg/mL propidium iodide the samples were analyzed on a FACSAria II (upgraded to III; BD, Heidelberg, Germany), as previously described [73]. Cells in X1, X2, and Xin gates were sorted in CMFH. After sorting, cells were lysed in Trizol.

### 2.4. Irradiation and RNAi

Planarians were γ-irradiated with 60 Gy in a Gammacell 40 irradiator (Best Theratronics, Ottawa, ON, Canada). After irradiation, animals were amputated into head, trunk, and tail fragments. Three or 6 days after amputation, tail fragments were collected for RNA extraction. For *SmB(RNAi)*, 5 mm long starved planarians were injected with double stranded RNA (dsRNA) in the gastro-vascular system using a Nanoject II (Drummond scientific, Broomall, PA, USA), with 3 pulses of 32 nl per day over 3 consecutive days. Animals were amputated 24 h after the last injection into head, trunk and tail fragments and the tail fragments were collected in Trizol at 6 dpa.

### 2.5. RNA Extraction and cDNA Synthesis

The RNA extraction was carried out with Trizol, following manufacturer’s instructions. Briefly, samples collected in 500 μL Trizol were vortexed for 5 s. The animals were incubated 5–15 min at RT (depending on the type and size of the samples), vortexed every 5 min. If fragments were not completely dissolved after 15 min incubation, an additional 500 μL of Trizol was added and the incubation repeated. Two-hundred μL of chloroform was added into the tube per 1 mL Trizol and shaken vigorously by hand for 10–15 s. Then, samples were incubated for 3 min at RT and centrifuged at 12,000× *g* for 15 min at 4 °C. The tube was carefully taken from the centrifuge and the upper aqueous phase was transferred into a new 1.5 mL reaction tube. Then, 500 μL isopropyl alcohol was added per 1 mL Trizol and the tube was shaken vigorously by hand for 10–15 s. The samples were incubated for 10 min at RT and then centrifuged at 12,000× *g* for 10 min at 4 °C. The supernatant was discarded, the pellet was washed with 1 mL 75% ice-cold EtOH and centrifuged at 7500× *g* for 5 min at 4 °C. The supernatant was discarded and the pellet was air-dried for a few minutes. The samples were resuspended in 10–20 μL RNase free water, depending on the pellet size. Total RNA concentration was measured with a Nanodrop and/or a Bioanalyzer. Total RNA was stored at −80 °C until use. Reverse transcription (RT) was carried out using the high-capacity cDNA reverse transcription kit (ThermoFisher Scientific, Dreieich, Germany) in 20 μL reaction volume, according to manufacturer’s instructions. Briefly, 250 ng of total RNA was used for RT, using Random Hexamers; MilliQ water instead of RNA was used as RT-control. After RH annealing (10 min at 25 °C), RT was carried out for 2 h at 37 °C.

### 2.6. RT-PCR and RT-qPCR

Gene expression was mostly assessed via quantitative PCR, with the exception of 20 candidate blastema transcription factors, whose expression was checked via conventional, semi-quantitative RT-PCR. Briefly, The TaqMan Fast Universal PCR master mix (ThermoFisher Scientific, Dreieich, Germany) was used, with custom designed Taqman probes. The reaction was carried out on ABIPrism 9600 HT with fast block, in 20 μL total volume. An initial denaturation step of 20 s was followed by 40 cycles with 95 °C for 1 s denaturation and annealing/elongation at 60 °C for 20 s. Transcript levels were normalized to the housekeeping gene *Gapdh* using the ∆∆Ct method. Each oligonucleotides set was tested for specificity and for the dynamic linear range prior to experiments.

Conventional RT-PCR was performed as follows. Briefly, 1× PCR buffer, 2 mM dNTPs, 1.5 mM MgCl2, 0.4 mM forward primer, 0.4 mM reverse primer, 250 ng cDNA template, 1.25 U AmpliTaq360 were mixed in a reaction volume of 25 μL. Each reaction was centrifuged briefly and then amplified with the following protocol: 5 min at 95 °C followed by 35 cycles of 30 s denaturation at 95 °C, 30 s annealing at 56–62 °C (depending on the annealing temperature of the primers), 1 min elongation at 72 °C and a final step of 7 min at 72 °C. Oligonucleotides for the amplification of the genes tested are listed in Table S1.

### 2.7. RNA-Seq

#### 2.7.1. cDNA Library Preparation

Before Preparing the cDNA library, rRNA depletion was performed. To deplete rRNA, RiboMinus™ Eukaryote Kit (ThermoFisher Scientific, Dreieich, Germany) for RNA-Seq was used according to manufacturer’s instructions; one or two rounds of depletions were performed. Then, RNA fragmentation was performed. To perform fragmentation of the whole transcriptome RNA, RNase III was used from the SOLiD™ Total RNA-Seq Kit (ThermoFisher Scientific, Dreieich, Germany), according to manufacturer’s instructions. To clean up fragmented RNA, the RiboMinus Concentration Module (ThermoFisher Scientific, Dreieich, Germany) was used. To quantify the yield of the fragmented RNA, either the Quant-iT™ RNA Assay Kit with the Qubit Fluorometer (Invitrogen, Waltham, MA, USA) or the RNA 6000 Pico Chip Kit with the Agilent 2100 Bioanalyzer (Agilent, Santa Clara, CA, USA) were used, according to manufacturer’s instructions. To construct the amplified whole transcriptome library, hybridization and ligation of RNA were performed with the components from the SOLiD™ Total RNA-Seq Kit. To perform reverse transcription with hybridized and ligated RNA, 10xRT buffer, dNTP mix, SOLiD™ RT Primers and ArrayScript™ Reverse Transcriptase were used. To purify the reverse transcribed cDNA, the MinElute PCR purification Kit (Qiagen, Hilden, Germany) was used according to manufacturer’s instructions. For size-based selection of the cDNA, Novex pre-cast gel products (ThermoFisher Scientific, Dreieich, Germany), 50 bp DNA Ladder and SYBR Gold nucleic acid gel stain was used according to manufacturer’s instructions. Using a clean razor blade, the gel plug corresponding to 150–250 nt cDNA was excised. To amplify the size selected cDNA, 10xPCR buffer, dNTP Mix, SOLiD™ 5′ PCR Primer, AmpliTaq DNA polymerase and SOLiD™ 3′ PCR Primer were used. The PCR was performed as follows: 95 °C for 5 min and 15 cycles of 95 °C for 30 s, 62 °C for 30 s and 72 °C for 30 s. To purify the amplified DNA, the PureLink™ PCR Micro Kit (ThermoFisher Scientific, Dreieich, Germany) was used according to the manufacturer’s instructions.

#### 2.7.2. PCR, Emulsion PCR and RNA-Seq Run

The RNA-seq run was performed on a SOLiD 4HT sequencer. Each library template was clonally amplified on SOLiD™ P1 DNA Beads via emulsion PCR, according to the Applied Biosystems SOLiD™ 4 System Templated Bead Preparation Guide (PN 4448378).

#### 2.7.3. RNA-Seq Data Primary Analysis

Sequencing post-processing was performed so to convert the reads into FASTA files. Reads were converted into CSFASTA format (i.e., a color space representation of FASTA format) by the SOLiD4 sequencing software at the end of the run. Data were then transferred manually to the analysis server using the ‘export’ function of the SOLiD web interface, according to manufacturer’s instructions. The individual library directories did not have a predictable prefix, so a custom shell script was run to produce a file containing a list of the experimental directories and to exclude reads with missing and unassigned barcodes.

### 2.8. RNA-Seq Analytical Pipeline

For mapping, we used a previously-assembled and Blast2GO-annotated *Schmidtea mediterranea* transcriptome assembly, trinity5, generated from Illumina reads using Trinity (see https://doi.org/10.1016/j.celrep.2014.12.018, accessed on 10 November 2021). Using this assembly, SOLiD reads were mapped to this transcriptome using the color-space function available in Bowtie, in combination with Tophat. Reads were subsequently normalised for read count and variance using the R package DESeq. Pairwise differential expression results were generated (comparing every sample with all others) including log2 fold changes and adjusted *p*-values (*padj*), applying a filter of *padj* < 0.3.

### 2.9. Whole Mount In Situ Hybridization, Fluorescent In Situ Hybridization and Immunohistochemistry

The protocol for whole mount in situ hybridization (WISH) was performed as previously described [74]. All steps have been performed under gentle shaking, if not otherwise specified. Briefly, planarians were killed in 2% HCl in 5/8 Holtfreter for 2 min on ice, fixed in 4% Formaldehyde for 20 min at 4 °C, post-fixed in MetOH (100%) for 2 h at 4 °C and bleached in 6% H2O2 in MetOH overnight using a bleaching lamp at RT. Animals were then rehydrated through a descending series of 75%, 50%, and 25% MetOH in 5/8 Holtfreter and finally incubated in PBTx for 30 min at 4 °C for each step. Permeabilization was performed by incubating the animals for 8–10 min in 20 μg/mL Proteinase K in PBTx at 37 °C. The reaction was stopped in 2% Glycine/PBTx for 10 min at RT and washing in PBTx for 1 min at RT. After a post-fixation using 4% PFA/PBTx for 1 h at 4 °C the animals were again rinsed in PBTx for 20 min at 4 °C and then incubated in 0.1 M TEA/PBTx for 15 min at RT. The following acetylation was performed by adding 0.25% acetic anhydrite, incubating for 15 min at RT, adding again 0.25% acetic anhydrite, incubating for a further 15 min at RT and rinsing the animals in PBTx for 5 min at RT. Prior to hybridization, samples were conditioned in 1:1 prehybridization solution/PBTx at RT for 10 min, and in prehybridization for 1 h at 56 °C. The riboprobes were diluted in hybridization solution to a final concentration of 0.01 ng/mL and denaturated at 70 °C for 10 min. Subsequently the samples were incubated with one gene-specific riboprobe for 60 h at 56 °C. Afterwards, fresh hybridization solution (without riboprobe) was replaced, followed by an stepwise dilution of the hybridization solution with 20× SSC, at 56 °C, each step lasting 40 min. Samples were then incubated in Maleic acid buffer (MAB, 100 mM maleic acid, 150 mM NaCl, 0.1% Triton-X 100, pH 7.5) for 20 min at RT and blocked in 10% horse serum MABT (Buffer II) for 1 h at RT. Antibody incubation (1:2000 anti-DIG-Fab fragments in Buffer II) was performed for 3 h at RT and the unbound antibodies were washed away by rinsing in MABT at 4 °C over-night. Each specimen was then conditioned in modified NTMT buffer (AP buffer, 0.1 M Tris-HCl pH 9.5, 0.1 M NaCl, 0.1% Tween) 3 times at RT for 10 min and then developed with 10% PVA AP buffer at RT in the dark. The clearing was done by rinsing in PBTx for 5 min at RT, fixation in 4% PFA/PBTx for 30 min at RT, rinsing in PBTx at RT and washing in 100% EtOH for 20 min at RT. After washing in 50% EtOH/PBTx and PBTx for 5 min at each RT, the animals were mounted on coverslips with VectaShield and imaged under either a fluorescent stereomicroscope (Nikon SMZ18, Düsseldorf, Germany) or a confocal inverted microscope (Zeiss LSM780, Jena, Germany).

For Fluorescent in situ hybridization (FISH), animals were processed as previously described [75] using tyramide signal amplification (Perkin Elmer, Solingen, Germany) according to the manufacturer’s instructions. Probes used for double-FISH were labeled with either digoxigenin (DIG) or dinitrophenyl (DNP) (Mirus DNP Labeling Kit, Mobitech GmbH, Göttingen, Germany). After incubation in anti-DIG-POD (poly, 1:100, Roche) or anti-DNP-HRP (1:100, Perkin Elmer) samples were washed in PBS/0.1% Tween 20 for 2 h and were developed with FITC-tyramide or Cy3-tyramide (Perkin Elmer, Solingen, Germany). The first color reaction was quenched with 1% H2O2 in PBS/0.1% Tween 20 for 45 min, followed by 10 min at 56 °C in 50% formamide/2×SSC/1% Tween-20. Oligonucleotides used for the synthesis of the riboprobes are listed in Table S2.

In case immunohistochemistry was performed, after an additional wash step in PBTx, blocking occurred in 1% BSA/PBTx for 2 h at RT and the primary antibody was incubated in 1% BSA/PBTx overnight at 4 °C. Rabbit-anti-PIWI1 antibody was used at a 1:5000 dilution. The next day 7 PBTx wash steps were performed for 1 h each at 4 °C. Subsequently, 0.5 μg/mL secondary antibody was incubated in 1% BSA/PBTx at 4 °C overnight. After washing 6 times with PBTx for 1 h each the animals were counterstained with 5 μg/mL Hoechst in PBTx for 2 h, mounted with VectaShield on a glass slide and imaged with a confocal microscope.

### 2.10. Gene Specific Knock-Down by dsRNA Injection

For dsRNA-mediated RNAi of blastema TFs, 5 mm long starved planarians were injected with gene-specific dsRNA (oligonucleotides used are listed in Table S2) in the gastro-vascular system using a Nanoject II (Drummond scientific, Broomall, PA, USA), with 3 pulses of 32 nl over 3 consecutive days. Control animals were injected with either dsRNA against GFP or water. Animals were either left intact or amputated 24 h after the last injection into head, trunk, and tail fragments, and imaged at 7 and 14 dpa.

### 2.11. Statistical Analysis

One-way ANOVA with Tukey post-hoc test, Kruskal–Wallis test with Dunn’s multiple comparison post-hoc test, two-tailed Students’ t-test and Fisher’s exact test were performed using GraphPad Prism 8.0 and 9.0 (GraphPad software, La Jolla, CA, USA). When variance was found between different sets of data, Welch’s correction was applied. Adjusted *p*-value in RNA sequencing data (*padj*) was calculated in DESeq2 package using Wald-test. Linear dimensionality reduction was achieved via Principal Component Analysis, using the function prcomp in R.

## 3. Results

### 3.1. Sample List Definition and Pre-Run Quality Check

**Figure 1:**
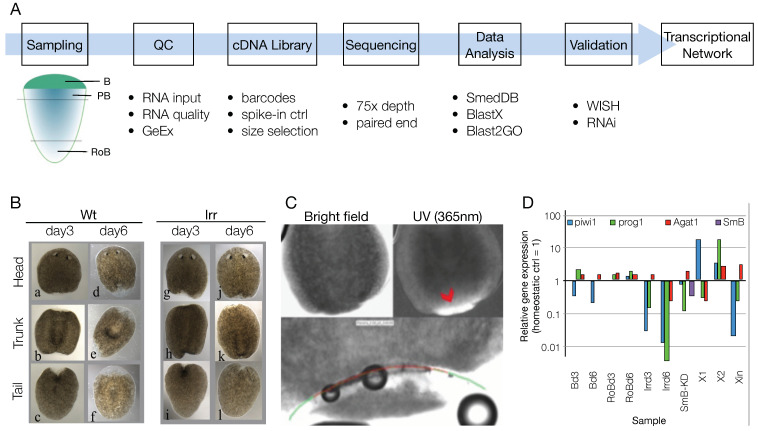
Experimental plan and sampling. (**A**) Schematics showing the experimental setup and the main techniques used to generate the shortlist of transcription factors involved in the anterior blastema regeneration. (**B**) Wild-type (a–f) and irradiated (g–l) animals 3 and 6 days after amputation in three fragments: head, trunk, and tail. (**C**) Day 3 wt trunk fragment under bright field and UV light illumination. The blastema is highly refractive under UV light, allowing the precise definition of the laser cut path (bottom). Red arrowhead: blastema-postblastema boundary; green line: laser cutting path (1st); red line: laser cutting path (2nd). (**D**) After RNA extraction and cDNA synthesis, the array of samples used in the study were assessed via qPCR for smedwi1, *prog1*, *Agat1*, and *SmB* (shown for *SmB*-KD sample only).

In order to study the transcriptional networks controlling the commitment and differentiation of the planarian SCs during regeneration, we generated RNA-Seq data from the anterior blastema at different time points of regeneration, and compared it with non-regenerating tissues (Figure 1A–C, Table S3). There are several sets of independently assembled *S. mediterranea* transcriptome data obtained via RNA-Seq (Table 1). In this study, we have strategically designed the RNA-Seq experiments to identify the transcriptional regulatory genes using multiple controls and an in silico data sorting strategy, to efficiently narrow down the number of candidate genes from the large RNA-Seq dataset (Figure 1A). Planarian regeneration lasts approximately two weeks but most of the missing tissues regenerate within one week from amputation. After amputation occurs, the SCs recruited at the wound site begin to proliferate and differentiate. Within approximately 2 days following amputation, a blastema forms, where progenitor and differentiating cells accumulate to regenerate the missing body parts [76,77]. Regenerating samples rich in progeny and differentiating cells were therefore collected at 3 and 6 days after amputation (dpa) (Figure 1B), separating the unpigmented blastema from the pigmented body part, either with a scalpel, or via laser microdissection (Figure 1C). Alongside the blastema samples, samples enriched in stem cells (i.e., X1 and X2 cell populations) and samples depleted of them, through different procedures, like irradiation (i.e., homeostatic animals at 3 and 6 days after irradiation), sorting (Xin cell population) and RNAi (i.e., *SmB* KD) were also collected. The bona fide non regenerating tissues at the posterior end of the fragment from which the blastema were separated (“rest of the body”, RoB) were also collected. Different amounts of total RNA from wild-type *S. mediterranea* homeostatic individuals (i.e., 50, 125, 250, and 1000 ng) were also processed, according to previous findings that showed no differences in gene detection for total RNA input exceeding 500 pg [78].

At the time this project was initiated, no methods were published on the use of small amount of planarian RNA for RNA-Seq, as was the case for the isolated blastema samples. Therefore, assessing the quality of the RNA was fundamental in order to deliver consistent RNA-Seq data. To collect enough total RNA, 30–40 blastema samples were pooled together, and the sample quality was checked by means of both spectrophotometry (Nanodrop, Bioanalyzer) and qPCR. The latter was important to confirm the identity of the samples (i.e., by checking the expression of known blastema markers) and rule out possible contamination from postblastema tissue. Marker genes were previously identified that helped to distinguish between blastema (devoid in stem cells) and postblastema [79]. Compared to homeostatic control, piwi1 was downregulated in blastema at both 3 and 6 days of regeneration, while *prog1* and *Agat1* were upregulated (Figure 1D and Figure S1). These results confirmed that the blastema samples did not contain stem but differentiating cells. Laser-dissected blastema at 3 dpa had a relatively high expression of piwi1, and therefore were kept separated from the mechanically dissected blastema in the downstream analyses (Figure S1). As expected, piwi1 was upregulated in both X1 and X2 samples; in X2 samples both *prog1* and *Agat1* were also upregulated, since this fraction contains both stem and progeny cells (Figure 1D and Figure S1). In Xin cells, piwi1 and *prog1* were downregulated while *Agat1* was slightly upregulated (Figure 1D and Figure S1). Irradiated samples showed low or no expression of piwi1 and *prog1* at both 3 and 6 days after irradiation; 6 days after irradiation *Agat1* was also downregulated (Figure 1D and Figure S1). In *SmB* RNAi samples, *SmB* downregulation was confirmed, together with piwi1 and *prog1* (Figure 1D and Figure S1).

### 3.2. RNA-Seq Data Quality Check Showed High Quality Scores and Confirmed the Upregulation of Known Markers of Cell Commitment and Differentiation in the Blastema Samples

After quality check, the samples were prepared for sequencing. Since polyA mRNA selection may introduce biases, especially for long transcripts [80], ribosomal RNA depletion was instead performed. In several samples, however, a single round of rRNA depletion was not enough (Figure S2A,B), therefore, a second round of rRNA depletion was performed that depleted rRNA below the level of detection of the Bioanalyzer (Figure S2C). The second round of rRNA depletion effectively reduced the number of reads that mapped onto rRNA sequences (Figure S2D).

After the cDNA library preparation, the barcoded samples were loaded onto 2 independent flow cells on the SOLiD4HT sequencer, where the quality of the RNA-Seq run is assessed in realtime. The distribution of the beads on the flow cells was homogeneous (Figure S3A) and the spectral purity (the separation of the signal in the 4 channels), a function of the intensity of single sequenced templates, was very high (Figure S3B), indicating that most of the beads contained a single cDNA species (monoclonal beads). We also assessed the quality per position along the sequencing reads, using FastQC to generate a box plot (a representative sample is shown in Figure S3C). This confirmed a very good quality, with an above average quality score up to position 39.

As samples with different amount of RNA were multiplexed, we compared the number of reads generated in each individual sample. Interestingly, in each of the two flow cells run, one sample was over-represented (intact_125 sample on FC1, RoB_M_d6_B in FC2 (Figure S4A). However, even in the least represented sample (intact_50) they generated more than 2 million reads, which guaranteed a coverage ≥ 75x. After combining the data from FC1 and FC2, a total of 1,009,885,098 reads were generated, of which only 3.2% were unassigned (Figure S4B). Except for the laser-dissected ones, blastema samples were represented by a minimum of 3,136,913 (Bla_d6_M_B) to a maximum of 45,626,513 (Bla_d3_M_B) reads (Figure S4C). These numbers guaranteed that virtually all transcripts were represented, also those with low level of expression, as transcription factors. We also included spike-in controls in some samples, in order to assess the linear range, for a reliable quantification. All the samples tested showed a broad linear range, spanning between 212 and 216 (Figure S5).

**Figure 2:**
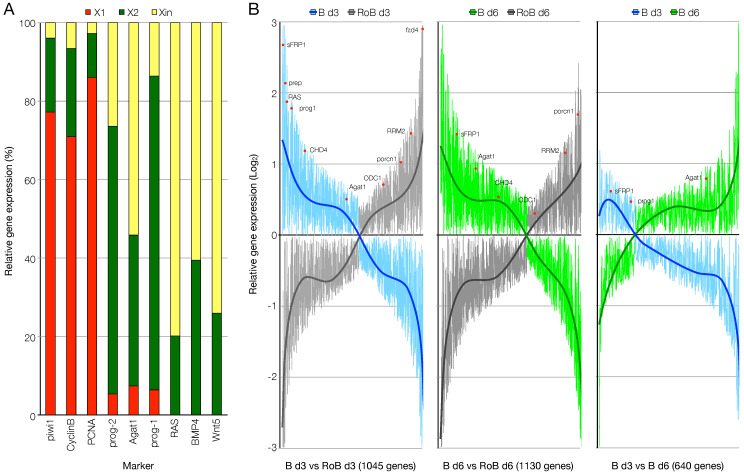
RNA-seq data relative to the expression of known genes. (**A**) Relative expression of genes representative of the FACS cell fractions in X1, X2, and Xin RNA-seq samples. (**B**) Genes differentially expressed between anterior blastema (**B**) and the respective non-regenerating rest of the body (RoB) at day 3 (left panel) and day 6 (center panel) of regeneration or between anterior blastema day 3 and anterior blastema day 6 (right panel), represented as butterfly charts. The position of some representative genes is provided (red dots). A threshold of 1.5-folds was applied, the trendlines represent the ratio of the expression of the genes considered between the pairs of samples shown.

After assessing the good quality of the generated data, we looked for the expression of known marker genes. In total, the expression of 44 selected planarian markers was evaluated, including housekeeping genes, stem cell markers, progenitor markers, and tissue specific markers (Figure S6A,B). Ubiquitous genes were found expressed in all samples, with *Gapdh* showing a very homogeneous level of expression (Figure S6A). Stem cell-specific markers as piwi1, PCNA, and CyclinB showed high expression in X1 samples (Figure 2A and Figure S6A,B). Progeny markers like *prog1* were found highly expressed in X2 samples, while *Agat1* was found expressed at similar levels in X2 and Xin cells (Figure 2A). Genes expressed by both stem and progeny cells, like Rb, p53, and *CHD4* were found enriched in X1, X2, and B samples (Figure S6B). Genes expressed by differentiated cells, like RAS, BMP4, and Wnt were found enriched in Xin samples (Figure 2A). As expected, the anterior pole marker *sFrp1* was found highly expressed in B day3 samples but not in RoB samples tissue (Figure S6A).

We then looked at the expression of known markers among the genes differentially regulated between B and RoB samples (1045 and 1130 genes at day 3 and day 6, respectively), or between B day 3 and B day 6 samples (640 genes). At both 3 and 6 dpa, the expression of post-mitotic progeny markers (*prog1*, *Agat1*) was higher in blastema samples, as it was the expression of the anterior identity markers (*Prep*, *sFrp1*). On the contrary, the expression of stem cell (*RRM2*), late progeny (*ODC1*) or terminally differentiated cell (*porcn1*) markers was higher in RoB samples (Figure 2B, left and central panels). The comparison between B samples at 3 and 6 dpa confirmed that while the early post-mitotic progeny marker *prog1* was enriched at 3 dpa, the late post-mitotic progeny marker *Agat1* was enriched at 6 dpa (Figure 2B, right panel).

### 3.3. An Ad Hoc Analysis Pipeline Sorted 65 Putative Transcription Factors Active in the Blastema

**Figure 3:**
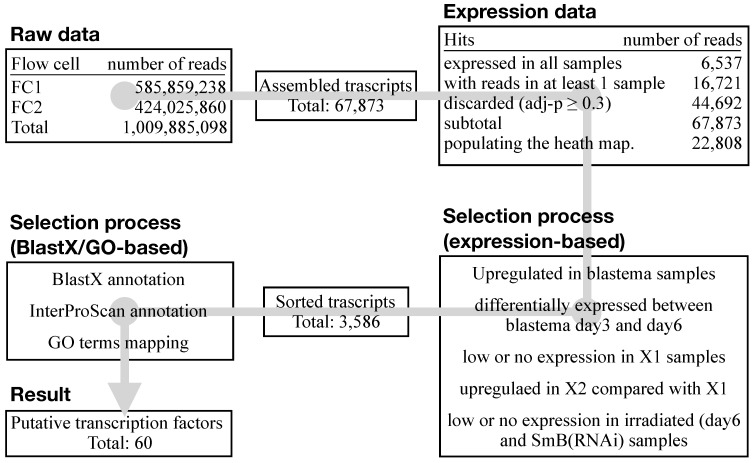
RNA-Seq analysis pipeline. Alongside the core analysis of the raw data (assembly, mapping, and annotation), the complete pipeline also consisted of data sorting. Putative transcription factors active in the blastema were selected according to their relative expression level in the considered samples and their functional annotation (Blastx, InterProScan and GO). A total of 60 putative blastema transcription factors were eventually shortlisted.

At the time the experiments were done, the *S. mediterranea* genome draft was fragmented and poorly annotated [50]. Therefore, using an in-house generated pipeline of scripts and open-source applications (e.g., Tophat, Bowtie), the SOLiD4HT RNA-Seq data were mapped on a transcriptome generated on 41bp single-end Illumina sequencing data [48]. In total, 1,009,885,098 reads were mapped to the newly generated transcriptome assembly, resulting in 67,873 assembled transcripts (Figure 3). For the comparison of RNA-Seq data among samples, the R/Bioconductor package DESeq was used to normalize read count and variance. Pairwise comparisons were performed with log2 fold changes and adjusted *p*-value. The data were then sorted based on the number of the read counts and the adjusted *p*-value in all samples, resulting in a final list of 22,808 transcripts (Figure 3).

**Figure 4:**
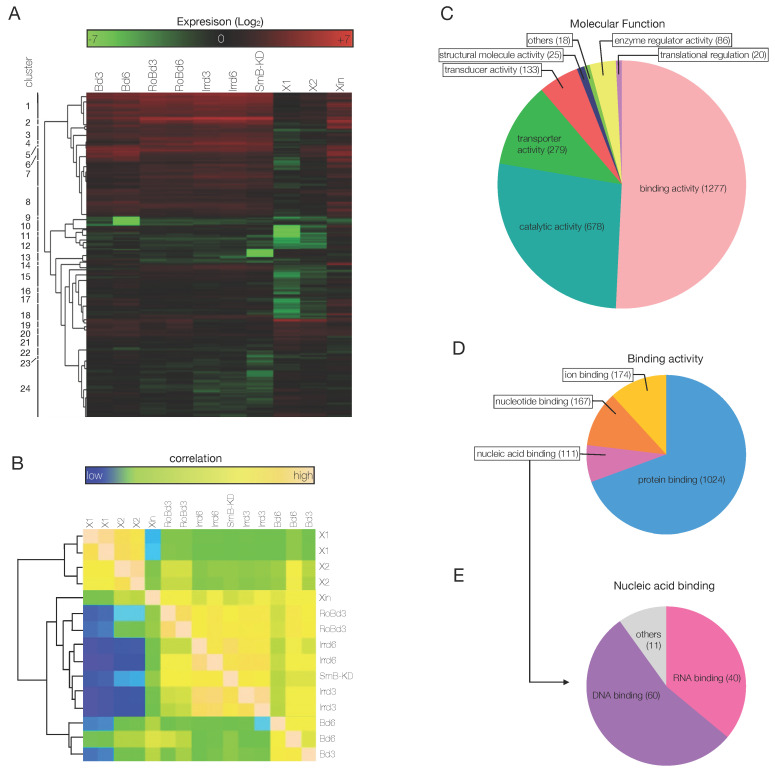
Sorting of the RNA-seq data. (**A**) A heat map was populated with 3586 genes that were sorted based on the differential expression among the samples, as explained in Figure 3. (**B**) The Pearson’s correlation based on the shortlist of 3586 genes showed how the samples analyzed in this study correlate expression-wise. Two main clusters of samples are visible: one that is stem cell-enriched (X1, X2 samples) and one that is non-stem cell-enriched (Xin, RoB, Irr d3, Irr d6, *SmB*(RNAi), blastema samples). Within the non-stem cell-enriched samples, the blastema samples clustered together in a sub-group (second most relevant hierarchical division). (**C**–**E**) The 3586 genes were grouped according to their molecular function (**C**). A lower hierarchical level of molecular functions was showed by the different classes of genes with binding activity (**D**). The pie chart showed how the 111 genes with nucleic acid binding activity distribute in the two main classes of nucleic acid binding: DNA- and RNA-binding (**E**).

Being interested in genes involved in cell differentiation, to shortlist interesting genes efficiently, the 22,808 transcripts were sorted again, by the comparison among the samples. The sorting process consisted in 4 sequential steps. First, transcripts upregulated in blastema samples compared to all other samples were selected. Second, since blastema is devoid of stem cells, only hits with no expression in X1 (or with an expression lower than in X2 and/or Xin) were selected. Third, hits not expressed in *SmB(RNAi)* fragments (since after amputation of *SmB* KD animals blastema formation is abolished) and irradiated (day 6) samples were selected. Fourth, genes differentially regulated between B day 3 and day 6 were also sub-clustered. After this expression-based sorting step, a total of 3586 putative transcripts were used to generate a heat map with 24 clusters (Figure 4A). In most of the clusters, genes were upregulated in the blastema samples compared to all other samples; however, in some of them they were downregulated (clusters 10, 12). Clusters 15–18 and 24 contained genes that were both heavily downregulated in X1 and X2 samples and differentially expressed between blastema at 3 and at 6 dpa. Genes in cluster 13 were undetectable in *SmB*(RNAi) samples, while downregulated but still detectable in irradiated samples (Figure 4A).

The shortlist of genes differentially expressed in the blastema was used to define the correlation among the samples used in the study. According to Pearson’s correlation, two main clusters of samples were individuated, one stem cell-enriched, which included X1 and X2 samples, and one differentiated cell-enriched, which included all other samples (Figure 4B). Within the second block, 2 distinct sub-clusters of samples were further defined: one that included Irradiated, *SmB*(RNAi), Xin, and RoB samples, and one that included the blastema samples only (Figure 4B). Consistent with the expected cellular composition, the highest correlation was observed between the SC-enriched fractions X1 and X2, consisting of stem, post-mitotic progeny, and small differentiated cells. Xin fraction, irradiated, and *SmB*(RNAi) samples are communed by the fact that they are all devoid of stem cells. Interestingly, in this correlation based on a restricted number of genes, RoB samples clustered together with the SC-depleted samples. Likely, this owes to the fact that RoB samples are *bona fide* non regenerating portions of tissue, thus they lack the array of post mitotic progeny cells that are on the contrary abundant in blastema tissues. Following the expression-based sorting of genes that we carried out, the absence of the progeny cells, rather than the relative presence of stem cells, is more determinant to cluster RoB samples together with SC-depleted samples. In a nutshell, the Pearson’s correlation proved that the queries applied to the RNA-Seq data to narrow down the number of differentially expressed genes considered for downstream analysis was successful and the shortlisted genes were strongly related to the molecular events that take place within the blastema.

In order to sort out the transcription factors from the list of 3586 putative genes, another sorting was performed, based on cross-species sequence alignment and on the recognition of conserved gene domains. A cross-species BLASTx analysis was first carried out, in which the nucleotide sequences of the putative transcripts were aligned to a cross-species protein database. After that, InterProScan and Blast2GO were used to classify the genes according to the presence of conserved domains and to predict the protein function, respectively. Eventually, the Blast2GO results were matched against the NCBI database of non-redundant invertebrate protein sequences, to assign the biological process, the molecular function and the cellular component to each one of the shortlisted genes, from the GO database [81]. Following this analysis, 1277 genes (the large majority of the genes for which a match in the aforementioned databases was found) had binding activity, 678 had catalytic activity, 279 had transporter activity, 133 had molecular transducer activity and 86 had enzyme regulator activity (Figure 4C). A second hierarchical classification based on molecular function was carried out for the 1277 genes with binding activity, which returned 1024 genes with protein binding activity, 174 with ion binding activity, 167 with nucleotide binding activity and 111 with nucleic acid binding activity (Figure 4D). Of the latter ones, following a third hierarchical classification based on molecular function, 60 were classified as DNA binding proteins and 40 as RNA binding proteins (Figure 4D). The 60 DNA binding genes were also analyzed for the biological processes they were involved Figure S7A) and for the cellular compartment to which they belong (Figure S7B).

During planarian regeneration, missing tissues are rebuilt by the cells that differentiate within the blastema. Differentiating cells are characterized by a deep cellular remodeling, increased metabolism, and the activation of the so-called developmental genes, which are necessary to leave the pluripotent state for the cell commitment. The most representative biological processes were cellular (44), metabolic (40), biological regulation (33), developmental (23), multicellular organismal (23), biogenesis (18), response to stimulus (18), and signaling (10) (Figure S7A). Unsurprisingly, since a large majority of the genes upregulated in the stem cell fractions were sorted out, the processes related to cell proliferation were under-represented (8) (Figure S7A). It is worth mentioning that one gene could be involved in more than one biological process, which explains why the total number of hits shown in the pie chart in Figure S7A (253) is higher than the number of the putative TFs (60). According to the cellular component annotation, 47 putative TFs localized to the cell, more specifically to organelles (39), macromolecular complexes (15) and membrane enclosed lumen (15) (Figure S7B).

In summary, the two sorting steps —one based on the differential gene expression among the samples used in the study and one based on the molecular functional annotations—produced a shortlist of 60 DNA binding genes. To these 60 candidate transcription factors, 5 additional ones were manually added, which the GO analysis failed to recognize, namely: *Ap2*, *Egr1*, and 3 genes of the Traf (TNF receptor-associated factors) family, which possess a zinc finger DNA binding domain as they act as signal transducers and transcriptional regulators.

### 3.4. The mRNAs of 44 Shortlisted Transcription Factors were Found Enriched in the Regenerating Blastema

Based on our RNA-Seq dataset, we identified 65 transcripts predicted to encode for DNA-binding proteins that were enriched in the anterior blastema of regenerating (day 3 and/or day 6) *S. mediterranea*. These putative transcription factors (Table S4) were named according to their best BLASTX homology hit, as described in the Materials and Methods. Thirteen genes were previously described in *S. mediterranea*, namely *Ap2*, *MyoD*, *Ets-1*, *Isl-1*, *Traf6*, *Fli1*, *Tcf15*, *Mitfl1*, *NF-YB*, *Prep*, *Fer3l-2* and two *FoxJ1*. Additionally, *ZicA*, identified in our RNA-Seq dataset, was previously published [61]. Five candidate genes were described in *S. polychroa*, 3 in *D. japonica* (*Musashi* and *Traf3*) and 7 in the *Schistosoma* genus (Figure S8). *Musashi* (mislabeled as a transcription factor) is expressed in the central nervous system (CNS) of *D. japonica* and regulates its regeneration [41]. *Ap2* and *Ets-1* are associated with regeneration initiation upon wound response in *S. mediterranea* [82]. *MyoD* is a master switch of myogenesis and is temporally and spatially segregated during *S. mediterranea* regeneration [29,30]. The LIM-homeobox gene *Isl-1* is required for the differentiation of Wnt-expressing cells at the posterior end of *S. mediterranea* [40]. *Tcf15* and the nuclear factor *NF-YB* are both involved in the regulation of pluripotency in *S. mediterranea*. [42,43]. *Prep* defines the anterior compartment during head regeneration [39].

Among the not yet described 44 candidate genes, 3 of them had no actual DNA binding activity, namely: *Pcbp3* (RNA-binding proteins), *Tufm*, and *Rnf11* (cytosolic proteins). Of the 41 remaining candidates, 15 were not transcription factors; they are either chromatin-associated proteins (e.g., *Histone 2A*, *Smc2*), chromatin remodeling factors (e.g., *Dsp1*, *EP300*, Jmjd2), topoisomerase (Top2) or signal transducers (e.g., *Traf3*, *Traf5*, *Traf6*). However, all are involved in the activation or repression of transcription, therefore we considered them as part of the transcriptional regulatory network that controls regeneration in the planarian blastema. Also, 4 genes were redundantly annotated. The details of the GO analysis of all the putative TFs considered in the study are summarized in Table S5. The resulting list of 40 candidate genes were independently validated by means of RT-PCR and WISH.

Since most of the candidate genes were selected based on their higher expression in blastema (either at 3 or 6 dpa) compared to the respective RoB, we expected to find a ratio between the expression of the genes in B and RoB samples >1. Thirty-three out of 40 tested genes and 11 out of 26 tested genes were found upregulated in B samples compared to RoB, at 3 (Figure S9A,B) and 6 (Figure S9C) dpa, respectively. Based on the gene expression, 3 groups of transcription factors were identified among the genes upregulated in the blastema: one with genes upregulated at both 3 and 6 dpa, one with genes upregulated at 3 dpa and neither up- nor downregulated at 6 dpa and one with genes upregulated at 3 dpa and downregulated at 6 dpa. In the first group we found *Otp*, *ZicA*, *Dr1*, *Ap2*, *Prep*, *Lhx2*, *Isl-1*, *Six-1*, *Irx3*, *Egr1*, and *Traf6*; in the second group we found *Tbx2/3*, *Hsf1*, *Nr4a2*, and *Tbx20*; in the third group we found *Traf3*, *Lmx1h*, *Gata123b*, *Ets-1*, *Zfp*, *MyoD*, *Traf5*, *Mitfl1*, *Rlm1*, *NF-YB*, and *Prdm1*.

**Figure 5:**
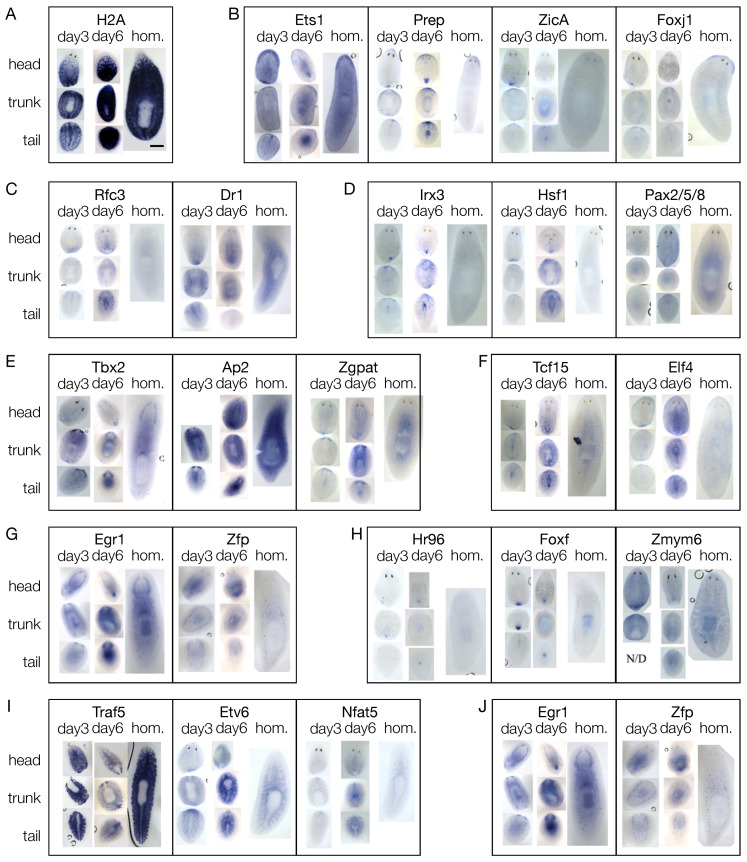
Expression of the shortlisted putative transcription factors in fragments at either day 3 or 6 of regeneration and in homeostatic animals, as for WISH. (**A**) SC-like patter of H2A. (**B**) *Ets-1*, *Prep*, *ZicA*, and *FoxJ1* were expressed in the region anterior to the photoreceptors. (**C**) *Rfc3* and *Dr1* were expressed along the midline. (**D**) *Irx3*, *Hsf1*, and *Pax2/5/8* were expressed only in regenerating fragments, either in the blastema or in other districts, but not in homeostatic animals. (**E**) Tbx2, *Ap2*, and *Zgpat* were expressed in the CNS. (**F**) *Tcf15* and *Elf4* were expressed in the mouth. (**G**) *Egr1* and *Zfp* were expressed in the pharynx. (**H**) *Hr96*, *FoxF*, and *Zmym6* were expressed in both mouth and pharynx. (**I**) *Traf5*, *Etv6*, and *Nfat5* were expressed in the intestine. (**J**) *Egr1* and *Zfp* were expressed in the testes. Scale bar: 1 mm.

In order to couple gene expression with morphology, we performed WISH against the candidate blastema transcription factors on regenerating fragments at 3 and 6 dpa, and on homeostatic animals (Figure 5 and Figure S10). Except for *Prdm1* and *Fer3l2*, all the genes studied were detected in the blastema, either at 3, 6, or 3 and 6 dpa. Many of them were also found expressed in other tissues, sometimes more than one (e.g., both in the pharynx and in the region anterior to the photoreceptors or both in the CNS and in the testes). As reported for *H2b*, also *H2a* showed a stem cell-like pattern (Figure 5A), like that of piwi1 (Figure S10A). Twelve genes were expressed in the region anterior to the photoreceptors, like *ZicA*, *Prep*, *FoxJ1*, and *Ets-1* (Figure 5B and Figure S10B). Fifteen genes were expressed along the midline, either dorsally or ventrally, like *Dr1* and *Rfc3* (Figure 5C and Figure S10C). Ten genes did not show a clear pattern of expression in intact animals, (i.e., they were expressed by dispersed sparse cells) but were upregulated in the blastema of regenerating animals, like *Irx3*, *Hsf1*, and *Pax2/5/8* (Figure 5D and Figure S10D). Nineteen genes were expressed in the CNS, like *Tbx2/3*, *Ap2*, and *Zgpat* (Figure 5E and Figure S10E). Altogether, 30 genes were expressed either in the mouth, like *Tcf15* and *Elf4*, or in the pharynx, like *Egr1* and *Zfp* or in both mouth and pharynx, like *Hr96*, *FoxF*, and *Zmym6* (Figure 5F,G and Figure S10F–H). Four genes were expressed in the intestine, like *Traf5*, *Etv6*, and *Nfat5* (Figure 5H and Figure S10I) and two were expressed in the testes, namely *Zfp*, and *Egr1* (Figure 5I and Figure S10J).

**Figure 6:**
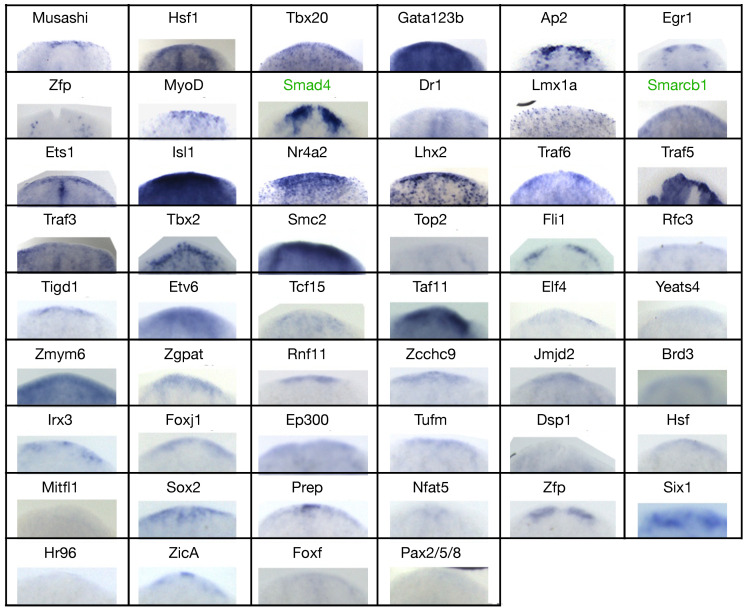
Expression of the shortlisted blastema transcription factors in day 3 blastema, as for WISH. Transcription factors expressed in the anterior blastema of tail fragments after 3 days of regeneration, except for *Zmym6*, which is shown in the anterior blastema of a trunk fragment. In green, two genes that are not transcription factors but have some transcriptional regulation activity (*Smad4* and *Smarcb1*).

Altogether, WISH data showed that some of the transcription factors are transiently expressed only in the blastema (TF-G1), while others are expressed by both cells in the blastema and cells in non-regenerating tissues (TF-G2). This could suggest that the TF-G1 are expressed by a subset of transient progenitor cells (e.g., *Irx3*, *Rfc3*, *Pax2/5/8*), while the TF-G2 are expressed by both progenitor and differentiated cells of a certain lineage (e.g., *Tbx2/3*, *FoxJ1*, *ZicA*, *Traf5*, *MyoD*). However, the TF-G2, which are more numerous, showed a blastema signal that associated to a specific tissue, as in the case of the genes expressed in the CNS (e.g., *Tbx2/3*, *Ap2*, *Fli1*), in the pharynx (*e.g.*, *Irx3*, *EP300*, *Six-1*) or in the intestine (e.g., *Traf5*, *Etv6*), while the TF-G1 showed a generic signal in the blastema that was not associated to a specific structure (Figure 6). Therefore, we hypothesized that the TF-G1 regulate genes involved in polarity and patterning, while the TF-G2 regulate tissue differentiation. This hypothesis is further supported by the fact that the TF-G1 were usually found expressed in regions that provide positional instructions, like the midline (e.g., *Dr1*) and the region anterior to the photoreceptors (e.g., *ZicA*). As we classified the blastema transcription factors according to their expression pattern, we also speculated about their derivation from a specific germ layer (Figure S11A). To experimentally confirm this, we performed double FISH experiments against markers specific for the three germ layers and the germ line. The tissue-specific markers used were porcupine (*porcn1*, for endodermal derivation [83]), prohormone convertase 2 (*PC2*, for neurectodermal derivation [84]), myosin heavy chain (*myhc*, for mesodermal derivation [85]), and the germinal histone 4 (*gH4*, for the germ line [86,87,88]).

**Figure 7:**
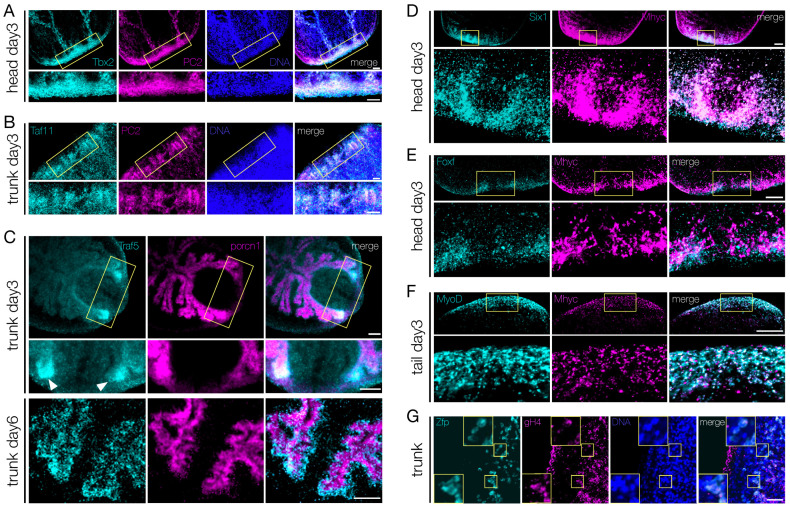
Co-localization of the transcripts of the shortlisted TFs with those of tissue-specific markers. (**A**,**B**) Co-localization of *Tbx2/3* (**A**) and *Taf11* (**B**) mRNAs with the pan-neuronal marker *PC2* (as for double-WISH) in day 3 blastema of regenerating head fragments. The areas in the upper panels surrounded with the yellow frames are enlarged in the respective lower panels. Scale bars: 50 μm. (**C**) *Traf5* expressing cells are located around the gut linen, which is marked by porcupine (as for double-WISH), both at day 3 (upper and middle panels) and at day 6 (lower panel) of regeneration. *Traf5* signal is stronger in the newly formed tissue close to the wound, especially in the early phase of regeneration (white arrowheads). The yellow frames in the upper panel show the regions enlarged in the middle panel. Scale bars: 50 μm. (**D**–**F**) Co-localizion of *Six-1* (**D**), *FoxF* (**E**), or *MyoD* (**F**) mRNAs with *Myhc* mRNA (as for double-WISH) in blastema at day 3 of regeneration. The areas in the upper panels surrounded with the yellow frames are enlarged in the respective lower panels. Scale bars: 50 μm. (**G**) Cells co-expressing *Zfp* mRNA with the germinal histone 4 mRNA, in the trunk fragment of planarians at day 6 of regeneration. Either double-positive or double-negative cells are visible. The inserts show an enlargement of the areas surrounded by the yellow frames. Scale bars: 50 μm.

Of the 44 transcription factors upregulated in the blastema, 19 were expressed in discrete neural populations throughout the CNS, in both homeostatic and regenerating planarians (Figure 5E, Figure S10E). We observed that virtually all the cells positive for *Tbx2/3* (Figure 7A), *Taf11* (Figure 7B), *Tbx20*, *Ap2*, *Traf6*, and *Fli1* (Figure S11B) were also *PC2*^+^. *Tigd1*, *Rnf11*, and *Zfp* were also found co-expressed with *PC2* (data not shown). *Porcn1*, expressed by the phagocytic cells in both homeostatic and regenerating intestine [89] is a marker for intestinal cells. Both *Traf5* and *Etv6* were found expressed mostly in the regenerating intestinal branches (white arrowheads in Figure 7C and Figure S11C). However, *Traf5* was not co-expressed with *Porcn1*, rather *Traf5*^+^ cells surrounded *porcn1*^+^ cells (Figure 7C, lower panel). In order to analyze the transcription factors expressed in muscle cells, we used *myhc* as a muscle cell-specific marker. Two different myosin heavy chain (*myhc*) genes have been identified in freshwater planarians. One is expressed in the muscle fibers of the pharynx, the muscles surrounding the gastro dermis, in a few scattered cells throughout the body-wall, and in some muscle fibers in the mesenchyme at the base of the pharynx, while the other *myhc* gene is expressed in the sub-epidermal body-wall musculature and in the dorsoventral fibers [30,90,91]. We found that *Pcbp3*, *Irx3*, *Six-1*, *Hr96*, and *FoxF* were expressed in the mouth and/or in the pharynx, while *MyoD* was expressed in sparse cells of the muscle body-wall, in the pharynx and at the edge of the blastema. Regardless of the gene-specific pattern of expression, all the novel transcription factors were found at least partially co-expressed with *myhc*. Most of the *Six-1*^+^ and *FoxF*^+^ cells found in the regenerating pharynx of head fragments at 3 dpa also expressed *myhc* (Figure 7D,E); a similar picture was observed for *Pcbp3* and *Hr96* in head and trunk posterior blastema at 3 dpa, respectively (Figure S11D, top and middle panels). Virtually all *MyoD*^+^ cells within the blastema of a tail fragment at 3 dpa were also positive for *myhc*; however, some *myhc*^+^ cells were *MyoD*- (Figure 7F). Interestingly, *Irx3*^+^ cells at the very edge of the blastema of tail fragments at 3 dpa were not *myhc*^+^, although in the rest of the blastema *Irx3*^+^ cells were also *myhc*^+^ (Figure S11D, bottom panel). Since among the transcription factors enriched in the blastema we found two that were also expressed in the testes (*Egr1* and *Zfp*; Figure 5J), we used *gH4* as germ cell marker to corroborate our observation. *Zfp* was found co-expressed with *gH4* in cells located in the dorsal-lateral area, in two stripes running from the neck backwards (Figure 7G). Remarkably, all other *gH4*^+^ cells found within the parenchyma (i.e., the stem cells) were *Zfp*-. A similar result was observed for *Egr1* (Figure S11E).

In order to better define the spatial/temporal expression of the genes under investigation and to prove their derivation from the SCs, we looked at the expression of the PIWI1 protein. Owing to the longer turnover of the protein, PIWI1 is found in both stem and progeny cells [82]. Shortly following amputation (18 hpa), the PIWI1^+^ cells that are found at the wound site are also piwi1^+^; however, from 48 hpa onwards, PIWI1^+^ cells found in the blastema are virtually all piwi1-. Immunohistochemistry targeting PIWI1 was performed after double FISH, so that the expression of the blastema TFs could be correlated with both that of tissue-specific markers and that of the PIWI1 protein. We found PIWI^+^ cells among the *Tbx2/3*^+^/*PC2*^+^ (Figure S12A, upper panel), *MyoD*^+^/*myhc*^+^ (Figure S12A, middle panel), *Six-1*^+^/*myhc*^+^ (Figure S12A, lower panel) and *Zfp*^+^/*gH4*^+^ (Figure S12B) cells. A similar picture was also found for other blastema TFs (*Gata123b*, *Ap2*, *Traf6*, *Fli1*, *Tigd1*, *Taf11* and *Rnf11*, *Pcbp3*, *Irx3*, *Hr96* and *FoxF*; data not shown). The co-expression of the blastema TFs with PIWI1 suggests that the blastema TFs are active in SC progeny that is enriched in the blastema and that will contribute to the regeneration of the missing tissues.

### 3.5. Shortlisted Blastema Transcription Factors Play a Role in Regeneration

**Figure 8:**
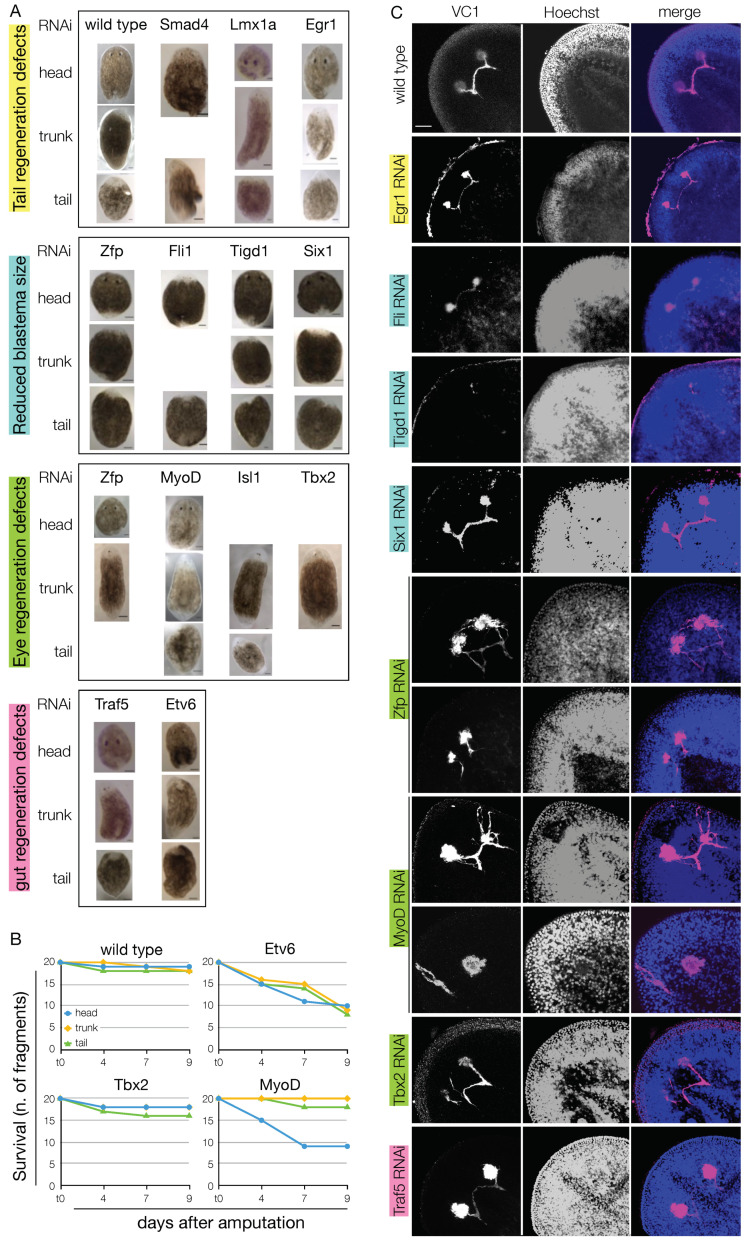
The knock-down of the blastema transcription factors resulted in regeneration defects. (**A**) Following dsRNA-mediated gene knock-down, most of the blastema transcription factors tested resulted in regeneration defects. The Knock-down of *Smad4*, Lmh1a, and *Egr1* resulted in tail regeneration defects (n = 7/9, 13/17, 17/24, respectively) and occasionally in eye regeneration defects (n = 3/9, 4/8, 20/43, respectively). The knock-down of *Zfp*, *Fli1*, *Tigd1* and *Six-1* resulted in a blastema of a reduced size, regardless of the fragment considered (n = 32/42, 41/53, 11/19, 16/23, respectively). The Knock-down of *Zfp*, *MyoD*, *Isl-1* and *Tbx2/3* resulted in eye regeneration defects (n = 21/23, 26/26, 10/10, 9/13, respectively). The Knock-down of *Traf5* and *Etv6* resulted in gut regeneration defects and the formation of body-wide lesions (n = 14/16, 20/20, respectively). (**B**) In spite of the regeneration defects displayed, most of the knocked-down genes did not alter significantly the lethality rate; exceptions to this rule were *Etv6*, with an average lethality of almost 50% at 9 dpa (n = 27/60) and *MyoD*, with a lethality rate >50% but limited to the head fragments (n = 48/60). (**C**) The immunostaining against arrestin (VC-1) showed that the knock-down of virtually all genes that produced tail, blastema or eye regeneration defects resulted also in the mis-projection of the visual neurons (e.g., *Zfp*, *MyoD*, Tbx2) or the reduced size of the photoreceptors (e.g., *Egr1*, *Fli1*, *Tigd1*, *Six-1*). Scale bars in C: 50 μm.

Whole mount in situ hybridization and immunohistochemistry data showed that virtually all the blastema TFs shortlisted from our RNA-Seq data are indeed enriched in the blastema (either at 3, 6, or both at 3 and 6 dpa). Also, the data showed that many are co-expressed with markers of differentiated tissues that derive from all the germ layers—including the germline—like *PC2*, *myhc*, and *gH4*. We also found that virtually all the blastema TFs co-express also the PIWI1 protein, suggesting that they are expressed by progeny cells. Next, we aimed to identify the function of 20 TFs in planarian, using dsRNA-mediated RNA interference (RNAi). Animals were injected three times on three consecutive days and then amputated into head, trunk, and tail fragments. The gene knockdown efficiency was assessed via qPCR after 4 or 7 days from the first dsRNA injection (Figure S13A). In most of the cases a reduction of the gene expression greater than 75% was observed. The regeneration process was assessed for macroscopic abnormalities after 7 and 14 dpa. Despite their effective knockdown, 7 out of 20 blastema TFs did not show any evident phenotype, namely *Tbx20*, *Smarcb1*, *Gata123b*, *Ets-1*, *Nr4a2*, *Traf3*, and *Traf6* (Figure S13B). The knockdown of the other 13 blastema TFs produced visible phenotypes, like tail regeneration defects (*Smad4*, *Lmx1a*, *Egr1*), reduced blastema (*Zfp*, *Fli1*, *Tigd1*, *Six-1*), eye regeneration defects (*Zfp*, *MyoD*, *Isl-1*, *Tbx2/3*), gut regeneration defects (*Traf5*, *Etv6*) or a combination thereof (Figure 8A). For some genes, these were previously described (*smad4* [92,93]; *Fli1* [8], *Six-1* [94] (in *D. japonica*; no phenotype after one round of RNAi), *MyoD* [95,96]). All these defects could be observed already at 7 dpa. The observed defects were rarely lethal, exceptions being *Etv6* and *MyoD* (the latter only in head fragments) (Figure 8B). More than 70% of *Lmx1h*(RNAi) and *Egr1*(RNAi) fragments showed tail regeneration defects (n = 7/9 and n = 17/24, respectively); in most of the cases, this was the asymmetric regeneration of the tail (Figure 8A). The RNAi of *Zfp*, *Fli1*, *Tigd1* and *Six-1* resulted in the formation of a smaller blastema, and a general delay in the regeneration of the missing tissues. However, apart from *Tigd1*, the KD of thesis genes did not result in permanent regeneration defects, suggesting that the specification/differentiation of the progenitor cells took place in a spatially, but not in a temporally correct fashion. On the contrary, Tidg1(RNAi) animals failed to regenerate the eyes, but showed no other visible morphological defects (Figure 8C). The RNAi of *Zfp*, expressed in the testes and in sparse cells in the early blastema, resulted in eye defects in more than 90% of the dsRNA-injected animals (n = 21/23). In virtually all *Zfp*(RNAi) animals, VC-1 immunostaining clearly showed aberrant axonal guidance and multiple times crossed optic chiasma (Figure 8C), as it was also the case after *Tbx2/3* KD. This TF is expressed in the whole CNS, and in other animal models its ortholog is known to be involved in the axonal guidance [97]. Interestingly, some TFs with an eye phenotype, like *MyoD* and *Zfp* were neither expressed in the eyes, nor in the CNS (at least, not at a detectable level). Certain muscle cells of the body wall, however, are involved in providing positional instruction; therefore, a cell non-autonomous effect like the observed eye phenotype could be the result of defects in the differentiation of the patterning muscle cells. Both *Traf5* and *Etv6* were expressed in the intestine, especially in the forming branches during regeneration (Figure 5I, Figure 7C and Figure S11C). Their KD resulted in a smaller blastema size, reduced intestinal branching and dorsal lesions (Figure 8A,B), as formerly observed as a consequence of other TFs expressed in the gastrovascular system, like *Nkx2.2* [98]. The lack of *Traf5*^+^ cells within the gut, suggests that this TF is expressed by pre-migratory intestinal progenitors, rather than terminally differentiated phagocytes, which derive from the stem cells surrounding the gut.

Taken together, these observations indicate that the many of the blastema TFs have a function during regeneration, and their lack of expression results in both cell autonomous (i.e., the stereotypical pattern of visual cells and/or the axonal growth defects) and cell non-autonomous (i.e., the axonal guidance and the general patterning defects) effects, likely depending on the progenitor cells that express them.

## 4. Discussion

This study aimed to uncover the transcriptional regulatory genes underlying anterior regeneration in the planarian *S. mediterranea*. To do so, we defined an experimental and analytical setup based on RNA-Seq whose goal was to shortlist transcription factors (TFs) mainly active in the regenerating blastema, either at 3, 6 or both 3 and 6 dpa.

After amputation, planarian stem cells (SCs) start proliferating in the region next to the wound; post-mitotic early SC progeny accumulate between the proliferating SCs and the wound, and a blastema becomes macroscopically visible at the wound site after 48 hpa. Here, the SC progenies differentiate into the missing tissues (Figure 1B). According to single cell RNA-Seq data, we know that planarian SCs is a heterogeneous population where pluripotent SCs (σ-neoblasts) are undistinguishable from multipotent, lineage-committed SCs (γ-, ν-, ζ-, Nb4-, Nb7-neoblasts, possibly more sub-types), unless destructive analysis is performed [33,34,35,36,37,38]. Whether regeneration starts from σ-neoblasts (naïve model), from lineage-committed SCs (specialized-SC model) or a combination of these two, we know that, within the anterior blastema, virtually all the cells, tissues and organs that make the planarian body are specified and differentiated, as all the missing structures regenerate there. Therefore, the anterior blastema should express, in a precise temporally- and spatially-defined way, all the transcriptional regulators that are necessary to generate all the planarian cell types anew.

We decided to analyze blastema at 3 dpa, since at this stage the blastema is largely devoid of proliferating SCs, and at 6 dpa, to add a second time point to the analysis in order to differentiate between early, transiently expressed TFs, (i.e., those regulating early differentiation of the SC progenies) and tissue-specific TFs.

### 4.1. The Sampling Strategy and the Ad Hoc Defined Analytical Pipeline Effectively Shortlisted Blastema TFs

A previous effort aimed to identify key regulators of head and tail regeneration in planarians was made [57], where the whole anterior and posterior regenerating fragments were used. In this work, we wanted to shortlist transcriptional regulators that are enriched in the anterior blastema. Therefore, alongside with blastema samples, we collected and sequenced several non-regenerating samples, including stem cell enriched (e.g., X1 cell population), progeny-enriched (e.g., X2 cell population) stem cell -depleted (Xin cell population, *SmB*(RNAi) planarians), and stem cell- and progeny-depleted (e.g., irradiated planarians) samples. Also, thanks to the pre-RNA-Seq validation, we could confirm that the individually dissected blastema were not contaminated with postblastema tissue (i.e., with proliferating SCs), by means of qPCR. In order to include earlier regeneration time-points, we also tried laser micro-dissection. Although the preliminary expression profile of the fixed, laser-dissected blastema were similar to the manually dissected ones, the primary analysis of sequencing data returned a much smaller number of reads, possibly biased owing to the nonlinear amplification. For this reason, we presented the data from the laser-dissected blastema, but did not include them in the analysis.

### 4.2. The Blastema TFs Are Active in the Specification and Differentiation of the Regenerating Structures, including the Cells Responsible for Body Patterning and Positional Instruction

Among the shortlisted blastema TFs we found 13 that were previously described as TFs involved in the differentiation of cells with specialized functions. Some of these, like, *FoxJ1*, *Fli1*, *Mitfl1*, *NF-YB*, and *Ap2* are involved in the maintenance or differentiation of tissue-specific cells [8,45,99,100,101]. Others, like *Isl-1*, *ZicA*, *Prep*, and *MyoD* are expressed by subsets of cells and are responsible of providing positional information for the correct body patterning [39,40,61,100]. In the same way, many of the blastema TFs are expressed in a tissue-specific fashion.

For example, both *Etv6* and *Traf5* are expressed in the intestine (Figure 5I, Figure 7C and Figure S11C), and their KD produced defects associated with the failure to regenerate the intestine. Interestingly, *Traf5* is not expressed by phagocytes, but in the external lining of the intestine, where SC differentiate in gut progenitor cells before entering the gastrovascular layer [25]. Therefore, it is possible that *Traf5* is expressed by the gut progenitor cells, which failed to terminally differentiate, and accumulate outside the intestine, instead of migrating inside it.

*Tbx2/3* is expressed in the CNS, both in the brain and in the ventral nerve cords, and where the pharynx connects with the intestine, but not in the eyes or the eye primordia (Figure 5E). During regeneration, intense *Tbx2/3* signal is visible in the anterior blastema (Figure 5E and Figure 7A). *Tbx2/3*(RNAi) animals showed eye regeneration defects. *Tbx2b* is known as regulating axonal extension posteriorly to the optic chiasm and towards the visual center of the brain in Zebrafish [97]. *Schmidtea mediterranea Tbx2/3* could also be involved in the axonal guidance of the visual neurons, and this could explain the different degrees of eye regeneration defects that we observed after its KD.

As we saw for previously described blastema TFs, a subset of the newly characterized blastema TFs are not expressed solely in one tissue (Figure 5, Figure 6 and Figure S10). Rather, they are more broadly expressed, suggesting that these TFs are involved either in the differentiation of multiple cell types or in the differentiation of non-tissue-specific cells, like those of the anterior organizer [61,102] or the muscle cells that provide positional instructions [29]. Therefore, it was not surprisingly that some of the blastema TFs, once knocked-down, were found to produce a cell non-autonomous phenotype.

A tissue-specific TF known to give cell non-autonomous phenotype is *MyoD*. Being involved in the differentiation of muscle cells that provides positional instruction to other cells, *MyoD*(RNAi) animals showed a smaller blastema, delays in regeneration patterning problems, as eye defects (Figure 8C), and death (Figure 8B). Another blastema TF that we selected for loss of function study was *Zfp*. *Zfp* is expressed in the testes (Figure 5G) but also in the blastema, by some sparse cells at 3 dpa (Figure 6). *Zfp*(RNAi) animals showed eye defects like aberrant axonal guidance and multiple times crossed optic chiasma (Figure 8C). We could not exclude its expression in eye precursors cells, as reported for *ovo* [14] and *nanos*, which shares with *Zfp* the expression in the testes [103]. In that case, the eye phenotype observed after *Zfp*(RNAi) could be a direct consequence of its reduced expression. However, differently to both nanos and sine oculis, *Zfp* is not expressed in any of the cells of the photoreceptors, and it is therefore possible that the eye phenotype was an indirect consequence of the disruption of patterning signaling.

However, it is also possible that the eye phenotypes observed after the KD of blastema TFs that are not expressed in the eyes could also owe to the fact that the downregulation of one cell specific transcription factor disrupts, in part or completely, the whole blastema transcriptional network, and this could prevent the transcriptional regulation of one or more TFs in the downstream cascade. This could eventually result in defects in the regeneration of tissues and organs where the KD TF is not actually expressed.

The finding that a number of the blastema TFs described are expressed in more than one tissue suggests a certain plasticity of the progenitor cells, that may retain the expression of a TF needed for a certain cell type even when their pathway leads towards another one, as shown in the case of the hematopoietic cells in human [104] and also postulated for planarians [105].

**Figure 9:**
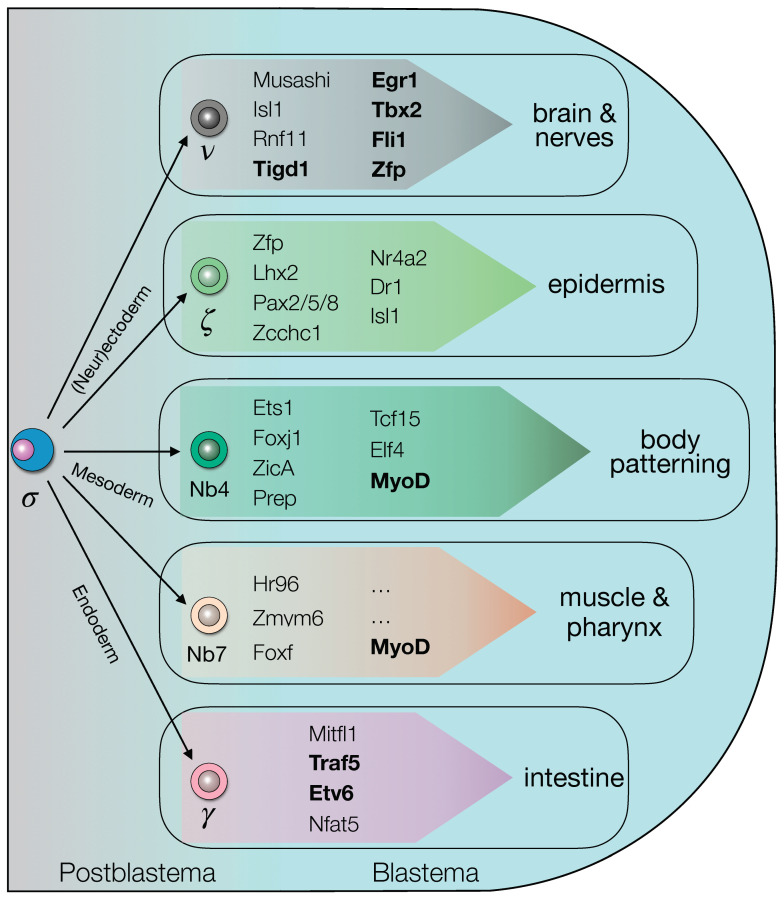
The blastema transcriptional regulatory genes landscape. The transcription factors expressed by the cells within the blastema could be either restricted to a specific lineage/tissue or not, as emerged from WISH and RNAi data. (functionally-validated genes are shown in bold).

Taken together our data indicate that the process of differentiation within the blastema, an essential step for the completion of regeneration, is regulated by a complex network of TFs, seemingly interdependent (Figure 9). The blastema TFs found in this study are involved in this process and could be useful tools to study early stage of planarian regeneration. To understand the microenvironment called niche surrounding stem cell differentiation and regeneration, collecting blastema tissue could give the advantage to uncover how tissue regeneration occurs in blastema tissue by intrinsic and extrinsic mechanisms. Understanding the mechanism of planarian regeneration, especially how stem cells differentiate into progenitors during regeneration, could improve pluripotency-based approaches for regenerative medicine.

## Figures and Tables

**Table 1:** Peer-reviewed articles presenting planarian transcriptome studies.

No.	Year	Author	Transcriptome Analysis	Species	Sequencing Samples
1	2010	Blythe, et al.	454, SOLiD3	*S. mediterranea*	Regenerating fragments at 6, 12, 24, 36, 48, 72, 96, 120, 144 h
					of anterior and posterior regeneration and intact
2	2010	Abril, et al.	454	*S. mediterranea*	Mixed sample of intact and regenerating planarians (1, 3, 5, and 7 days),
					Irradiates intact and regenerating animals (1, 3, 5, and 7 days of regeneration)
3	2011	Qin, et al.	Illumina HiseqTM 2000	*D. japonica*	Regenerating planarian from Day 1 to Day 10 and intact
4	2011	Adamidi, et al.	454, Illumina GAIIX	*S. mediterranea*	Whole animal
5	2011	Sandmann, et al.	Illumina Genome Analyzer II	*S. mediterranea*	Regenerating head from 0 to 3 days
			SOLiD3	*S. mediterranea*	Regenerating head and tail regions at 0, 1, and 6 h after amputation
6	2012	Solana, et al.	SOLiD4	*S. mediterranea*	Iraddiate animals at 2, 4, 7 days and wild type intact
7	2012	Shibata, et al.	HiCEP	*D. japonica*	Intact, Irradiated planarians, Neoblasts from X1/2 fractions
8	2012	Galloni, et al.	DGE	*S. mediterranea*	Irradiated and normal regenerating samples at 0–7 days after amputation
9	2012	Resch, et al.	Illumina GAIIX	*S. mediterranea*	non-irradiated or irradiated
10	2012	Nishimura, et al.	Sanger Sequences	*D. japonica*	Head fragments after amputation
11	2012	Lapan, et al.	Illumina Genome Analyzer II	*S. mediterranea*	Eyes, amputated heads (above the pharynx and coronal amputation was made to
					remove dorsal tissues including eye)
12	2012	Labbe, et al.	Illumina HiSeq 2000	*S. mediterranea*	FACS sorted stem cells, irradiate animals at 7 days post irradiation
13	2012	Onal, et al.	Illumina Genome Analyzer II	*S. mediterranea*	X1, X2, Xin
14	2013	Sikes, et al.	Illumina Sequencing	*Procotyla fluviatills*	Fragments of worms, intact
15	2013	März, et al.	Illumina HiScanSQ	*S. mediterranea*	Smed-pitx RNAi and control fragments at 3 days post amputation
16	2013	Kao, et al.	SOLiD3	*S. mediterranea*	Regenerating head and tail fragments 0, 6, 12, 24, 46, 48, and 72 h after amputation.
			454	*G. tigrina*	Tail fragments of Smed-*Prep* RNAi animals 24 h after amputation
				*Hofstenia miamia*	Embryonic development (2-cell stage through nine days old embryos from multiple parents)
17	2014	Srivastana, et al.	454	*S. mediterranea*	Regeneration at 1, 6, 18, 24, 72 h after amputation
18	2014	Vogg, et al.	Illumina HiScanSQ	*S. mediterranea*	FoxD RNAi tail stumps at 0 and 3 days post amputation and regenerating control
19	2014	Scimone, et al.	Illumina HiSeq	*S. mediterranea*	X1 isolation after amputation
20	2015	Reuter, et al.	Illumina HiScanSQ	*S. mediterranea*	dsRNA notum, 18 h post amputation
21	2015	Wheeler, et al.	Illumina HiSeq 2000	*G. tigrina*	Amputation, +serotonin, control
22	2016	Pang, et al.	Illumina HiSeq 2000	*D. japonica*	Whole animal
23	2016	Molinaro, et al.	Illumina HiSeq 2500, Single Cell Seq	*S. mediterranea*	FACS sorted X1, X2 from head
24	2018	Almazan, et al.	Illumina HiSeq 2500	*G. dorotocephala MA-C2*	Intact, 1, 4, days post amputation, Auricles
25	2018	Zeng, et al.	Illumina HiSeq 2500, scRNA-seq	*S. mediterranea*	Bulk RNA-seq of live cells and fixed cells (X1, X2, Xin), X1 neoblasts (200,000 cells) from
					wild-type animals, X1 + X2 cells from 7 day sub-lethally irradiated animals
26	2018	Mihaylova, et al.	Illumina NextSeq	*S. mediterranea*	FACS sorted G2/M cells from knockdown and control RNAi animals 3days of regeneration
27	2018	Ross, et al.	HiSeq 2000	*S. mediterranea*	SoxB1-2 RNAi and control RNAi animals at day 6, 14, and 24 after the first RNAI treatment
			scRNA-seq (Molinaro, et al., 2016)		
28	2019	Sekii, et al.	Illumina HiSeq 2000	*D. ryukyuensis*	Asexual sample, Sexual sample, Innate Sexual sample
29	2020	Forsthoefel, et al.	Nextseq 500	*S. mediterranea*	LCM (medial intestine, lateral intestine, non-intestine)

## Data Availability

All data presented in this study are available upon request.

## Supplementary Materials

The following are available online at https://www.mdpi.com/article/10.3390/biom11121782/s1, Figure S1: RNA-seq samples quality check via qPCR of all replicates used in the study. Figure S2: Effectivity of two rounds of rRNA depletion and distribution of reads mapping on rRNA sequences after RNA-seq. Figure S3: RNA-seq data quality check. Figure S4: Sample-wise RNA-seq reads distribution in the two flow cells used in this study. Figure S5: Analysis of the spike-in control added to the samples run in FC1. Figure S6: RNA-seq data relative to planarian genes for which a pattern of expression is known. Figure S7: Additional gene ontology (GO) annotations. Figure S8: Species distribution of 65 candidates. Figure S9: The expression of 40 transcription factor/transcriptional regulator genes found enriched in blastema samples. Figure S10: Expression of the shortlisted putative transcription factors in fragments at either day 3 or 6 of regeneration and in homeostatic animals, as for WISH. Figure S11: The blastema transcription factors are co-expressed with markers of cells from either neurectoderm, mesoderm, endoderm or the germ line. Figure S12: The blastema transcription factors are co-expressed with the PIWI1 protein. Figure S13: dsRNA-mediated RNA interference of the blastema TFs. Table S1: Oligonucleotides used for RT-PCR and RT-qPCR. Table S2: Oligonucleotides used for the synthesis of the riboprobes. Table S3: List of samples used for RNA-seq. Table S4: Top Blastx hits of the shortlisted candidates. Table S5: GO analysis of the shortlisted, putative TFs.
