# Supplementary material for: Transcription Factors Active in the Anterior Blastema of Schmidtea mediterranea
Source: Biomolecules. 2021 Nov 28;11(12):1782. doi: 10.3390/biom11121782 (PMC8698962; doi:10.3390/biom11121782)
Supplement: Supplementary file 1 [file biomolecules-11-01782-s001.zip › FigureS1.pdf]

**Supplemental figure 1**

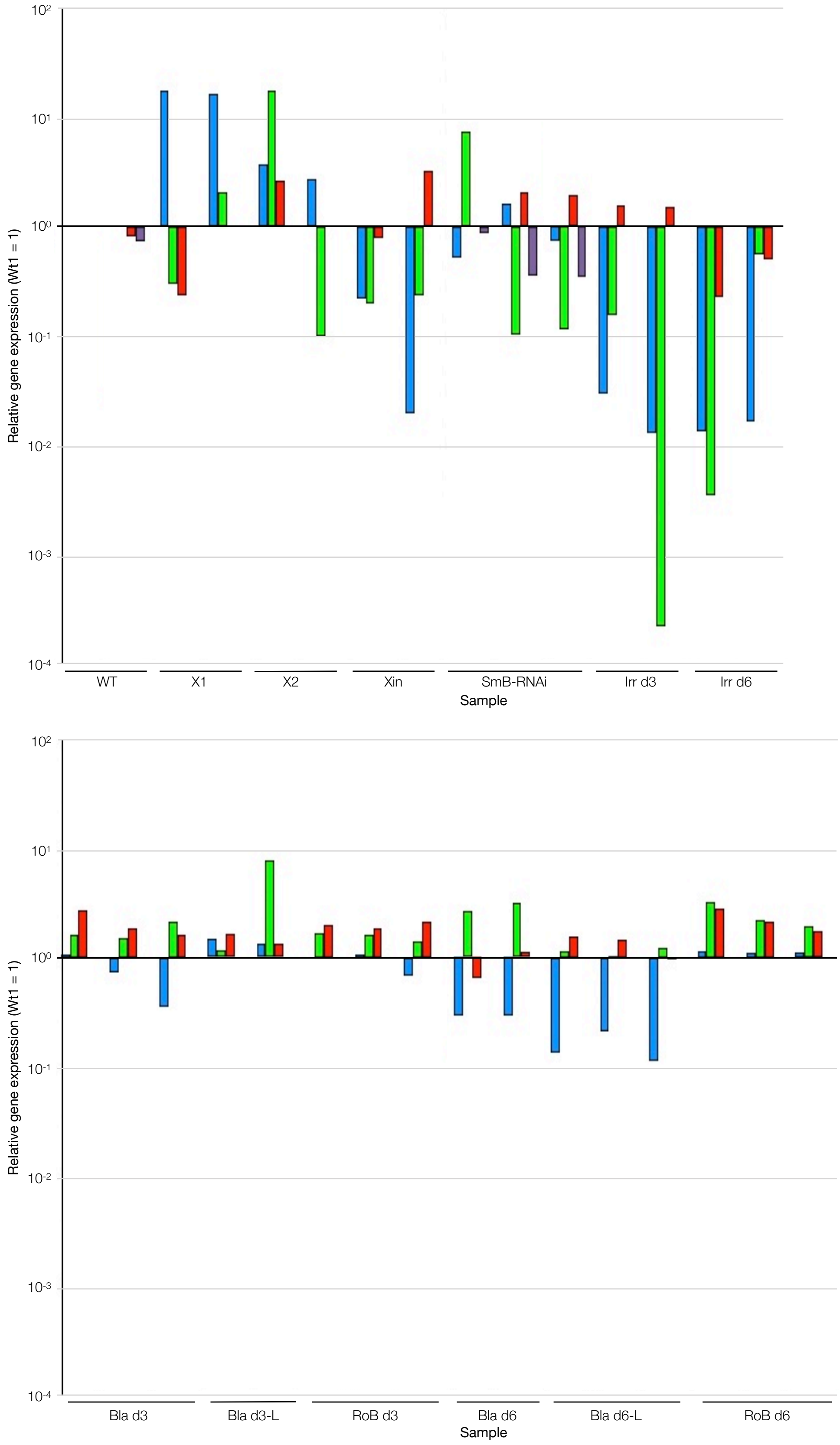


**Supplemental figure 1. RNA-seq samples quality check via qPCR of all replicates used in the study.** (**A**) After RNA extraction and cDNA synthesis, the array of samples used in the study were assessed via qPCR for smedwi1, NB.32.1g, Agat1 and smed-SmB (WT and SmB-RNAi samples only)
