# Supplementary material for: Transcription Factors Active in the Anterior Blastema of Schmidtea mediterranea
Source: Biomolecules. 2021 Nov 28;11(12):1782. doi: 10.3390/biom11121782 (PMC8698962; doi:10.3390/biom11121782)
Supplement: Supplementary file 1 [file biomolecules-11-01782-s001.zip › FigureS10.pdf]

Supplemental figure 10

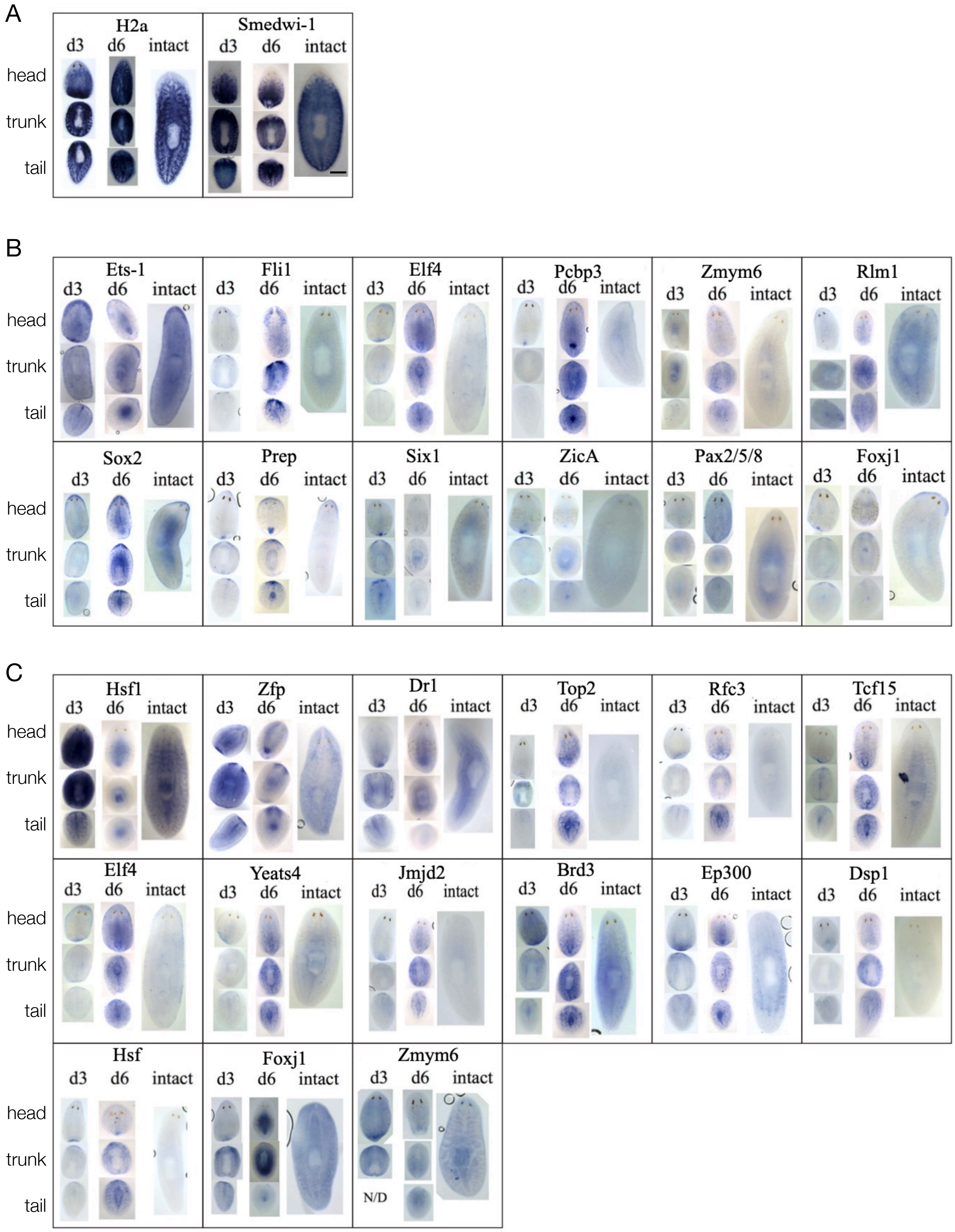

Supplemental figure 10 (continued)

D

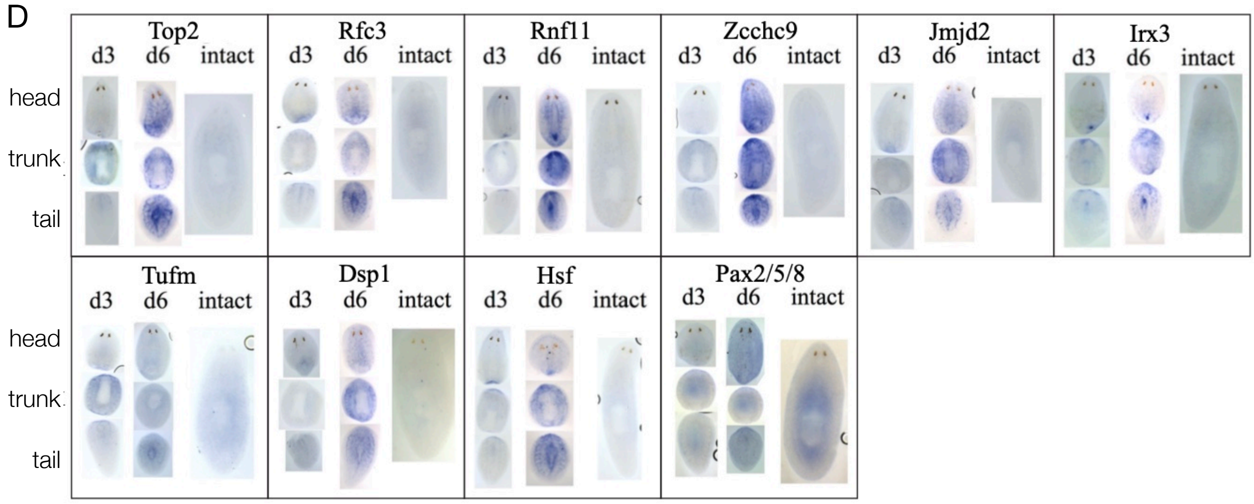

E

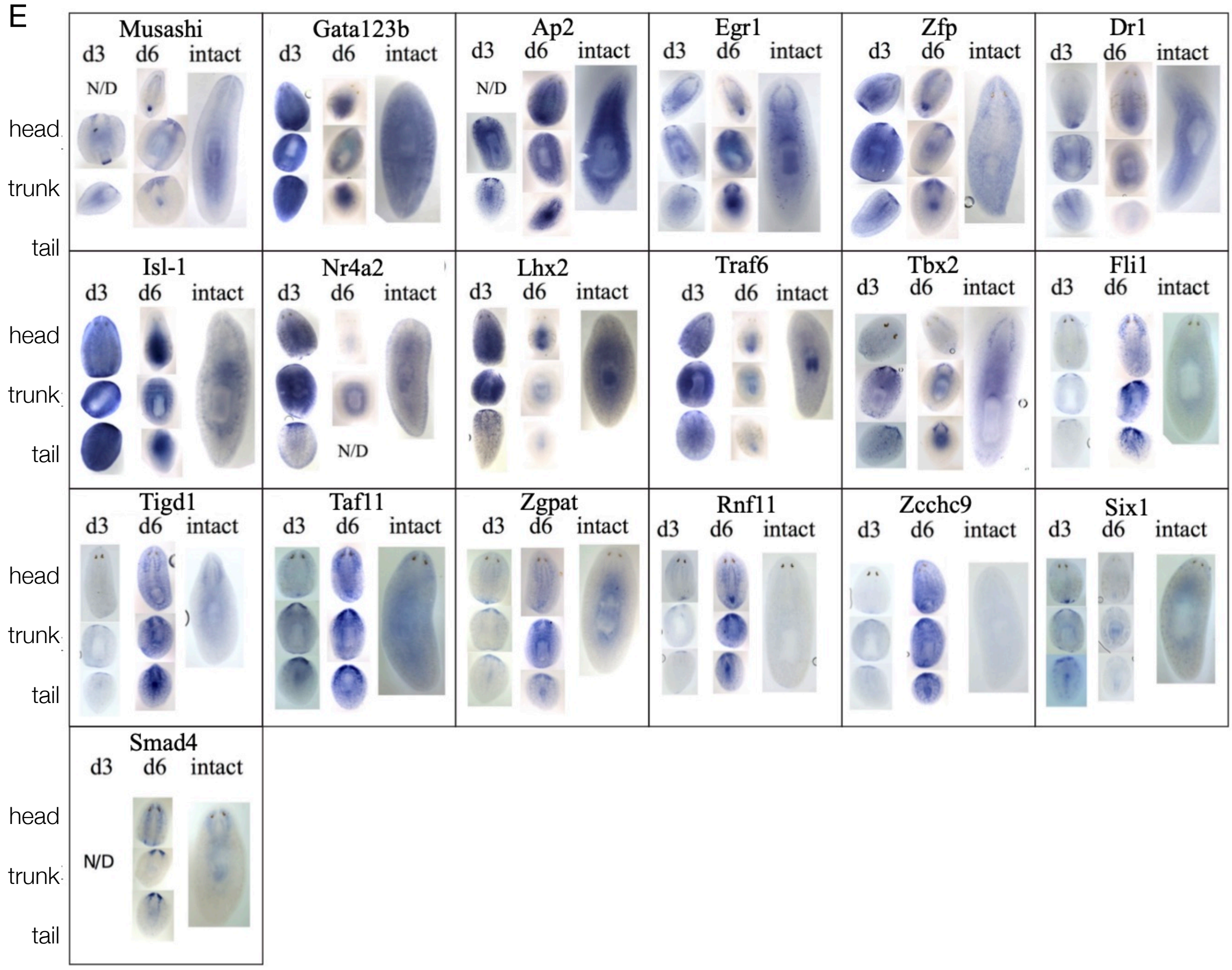

F

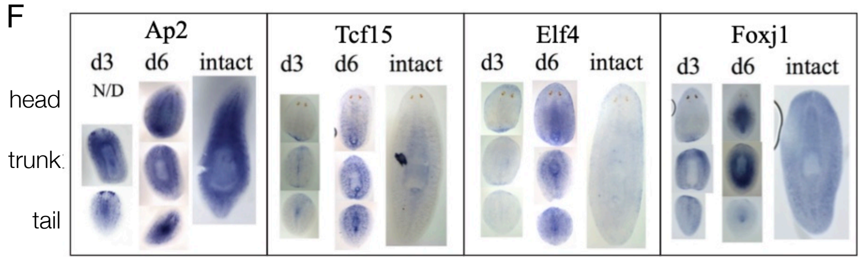

Supplemental figure 10 (continued)

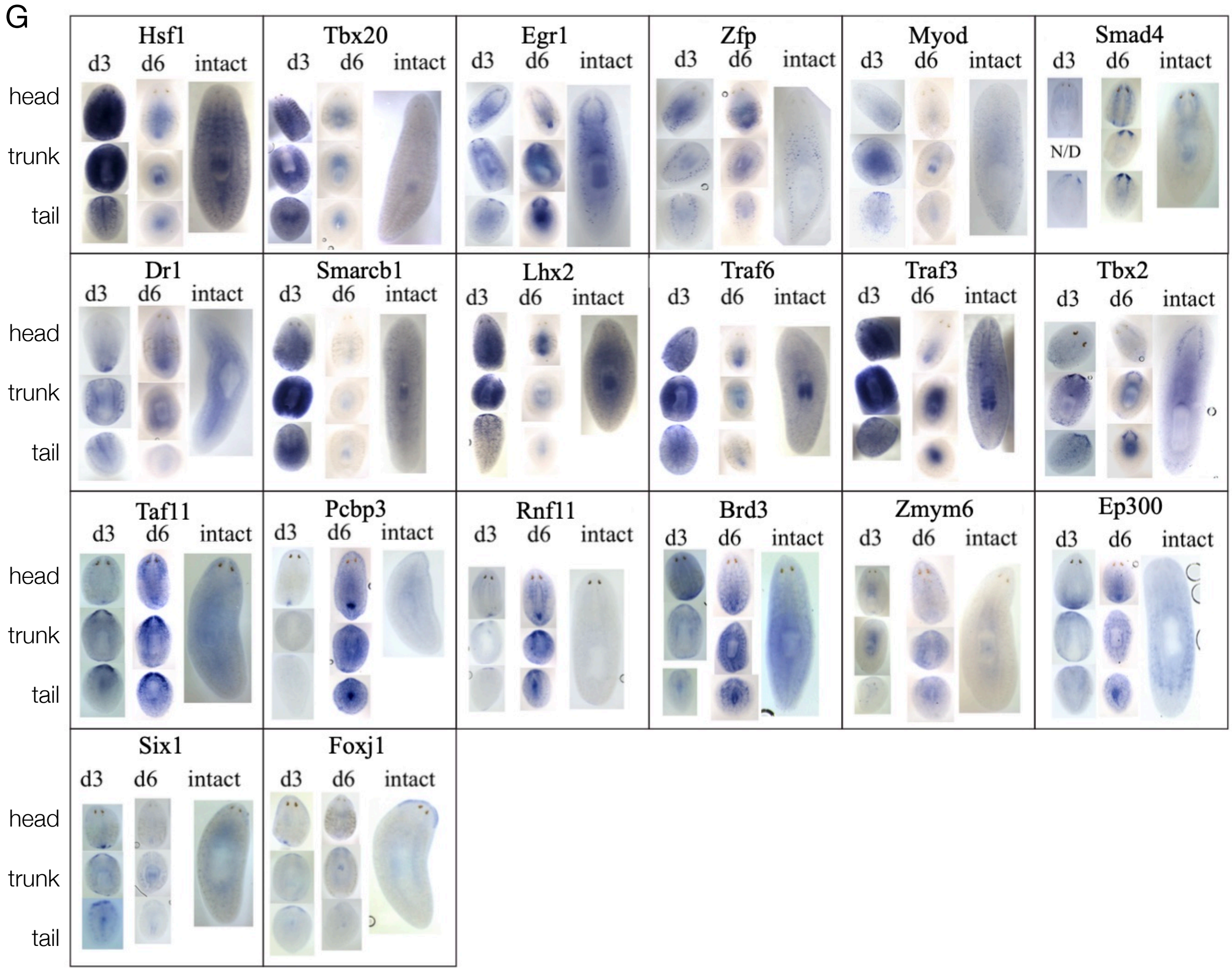

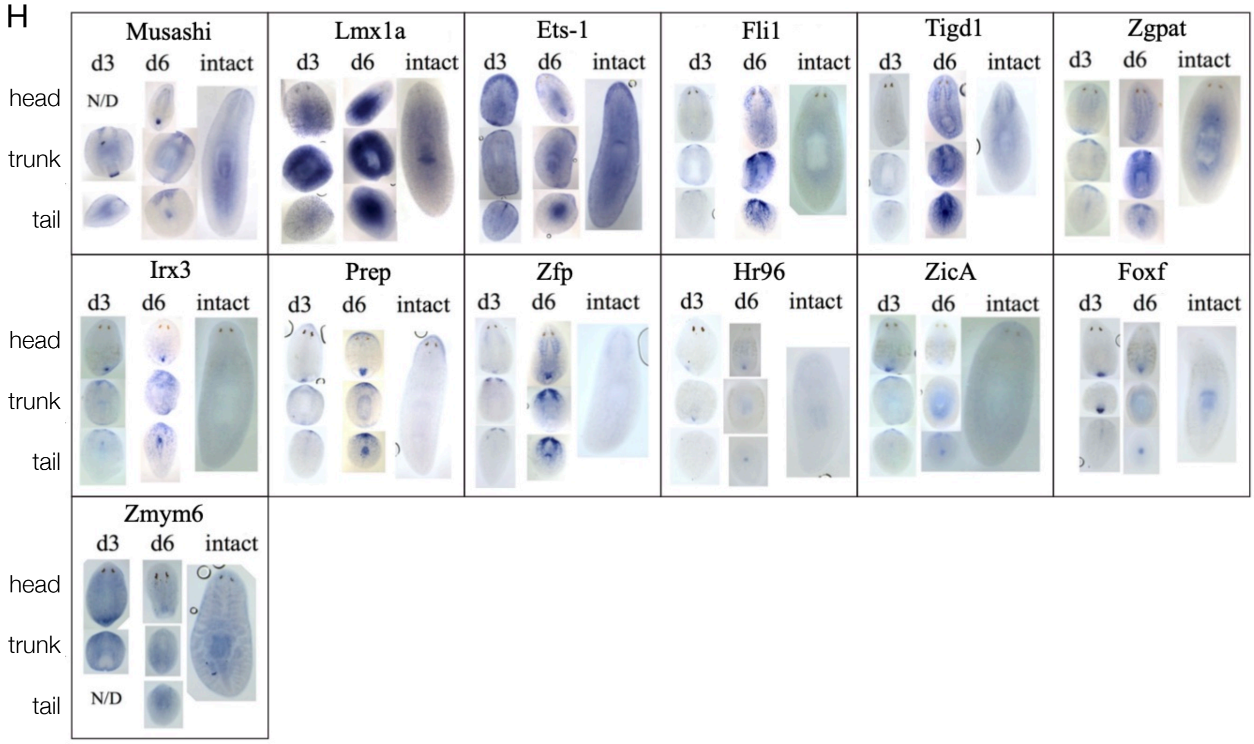

Supplemental figure 10 (continued)

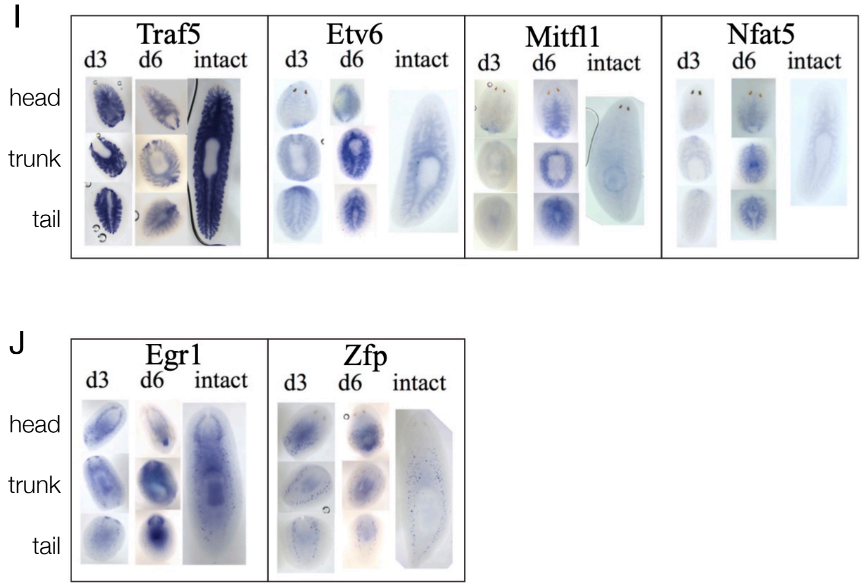

Figure 21. Candidate genes validation and expression pattern analyzed by WISH at 3 and 6 dpa and in homeostatic animals. (**A**) Stem cells like pattern of H2A expression (piwi-1 shown as reference). (**B**) Genes expressed in the region anterior to the eyes (12 genes). (**C**) Genes expressed along the midline (15 genes). (**D**) Genes expressed only in regenerating fragments and not in homeostatic animals (10 genes). (**E**) Genes expressed in the CNS (19 genes). (**F**) Genes expressed in the mouth (4 genes). (**G**) Genes expressed in the pharynx (20 genes). (**H**) Genes expressed in both mouth and pharynx (13 genes). (**I**) Genes expressed in the gut (4 genes). (**J**) Genes expressed in the testes (2 genes).
