# Supplementary material for: Transcription Factors Active in the Anterior Blastema of Schmidtea mediterranea
Source: Biomolecules. 2021 Nov 28;11(12):1782. doi: 10.3390/biom11121782 (PMC8698962; doi:10.3390/biom11121782)
Supplement: Supplementary file 1 [file biomolecules-11-01782-s001.zip › FigureS11.pdf]

Supplemental figure 11

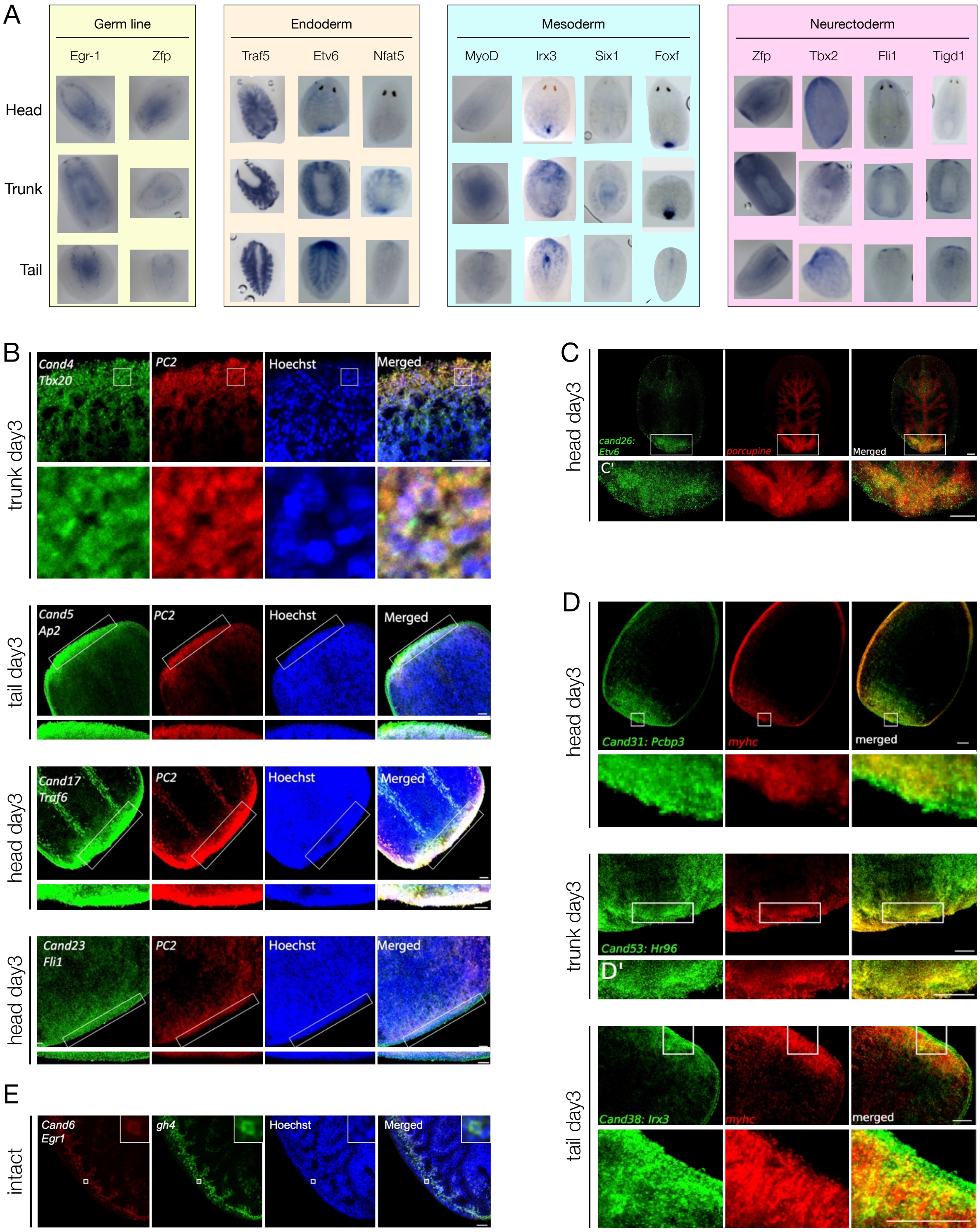

Supplemental figure 11. The blastema transcription factors are co-expressed with markers of cells from either neurectoderm, mesoderm, endoderm or the germ line. (**A**) Based on the pattern of expression, the blastema transcription factors were grouped according to their putative derivation from the three germ layers and the germ line. (**B**) Double FISH of the blastema transcription factors found expressed in the CNS with the pan-neuronal marker PC2. (**C**) Double FISH of the blastema transcription factors found expressed in the intestine with the gut cell marker porcn1. (**D**) Double FISH of the blastema transcription factors found expressed in the pharynx or in the muscle layers with the muscle cell marker myhc. (**E**) Double FISH of the blastema transcription factors found expressed in the testes with the germ cell marker gH4. Scale bars: 50 µm.
