# Supplementary material for: Transcription Factors Active in the Anterior Blastema of Schmidtea mediterranea
Source: Biomolecules. 2021 Nov 28;11(12):1782. doi: 10.3390/biom11121782 (PMC8698962; doi:10.3390/biom11121782)
Supplement: Supplementary file 1 [file biomolecules-11-01782-s001.zip › FigureS12.pdf]

Supplemental figure 12

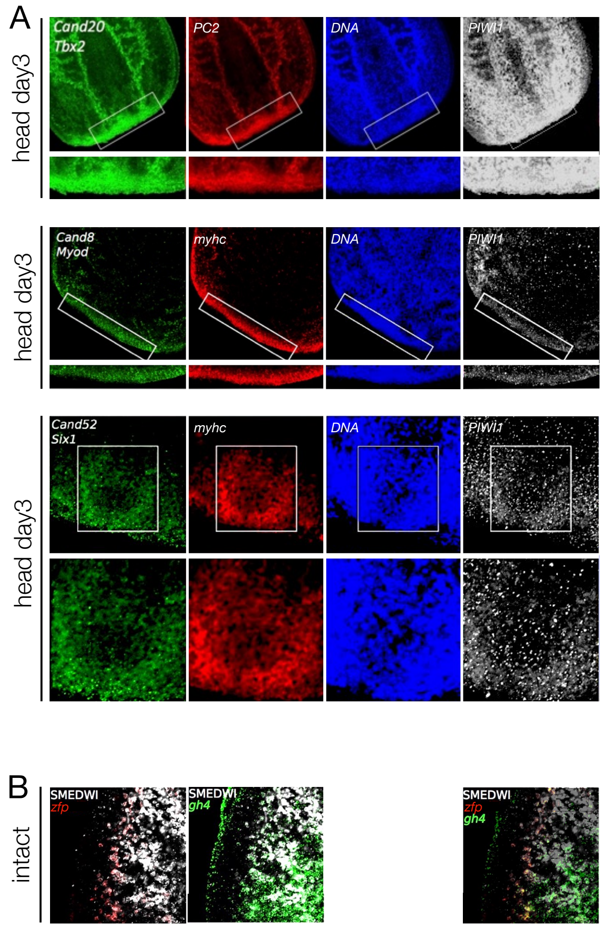

Supplemental figure 12. The blastema transcription factors are co-expressed with the PIWI1 protein. (**A**) (**B**) Double FISH of some representative blastema transcription factors with their respective tissue-specific markers in head fragments at 3 dpa; immunostaining against PIWI1 was carried out after FISH. From top to bottom: Tbx2 and PC2; MyoD and myhc; six1 and myhc. (**B**) Double FISH of zfp with the germ cell marker gH4 in homeostatic planarian; immunostaining against PIWI1 was carried out after FISH. the Scale bars: 50 μm.
