# Supplementary material for: Transcription Factors Active in the Anterior Blastema of Schmidtea mediterranea
Source: Biomolecules. 2021 Nov 28;11(12):1782. doi: 10.3390/biom11121782 (PMC8698962; doi:10.3390/biom11121782)
Supplement: Supplementary file 1 [file biomolecules-11-01782-s001.zip › FigureS13.pdf]

Supplemental figure 13

A

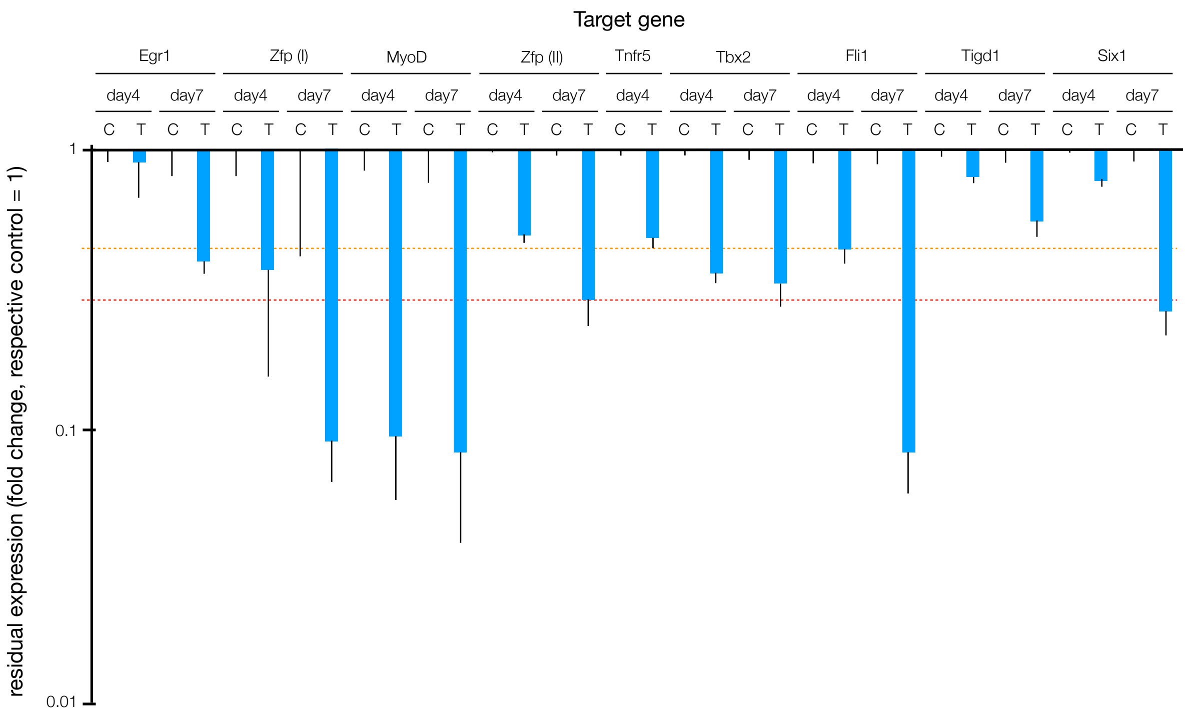

B

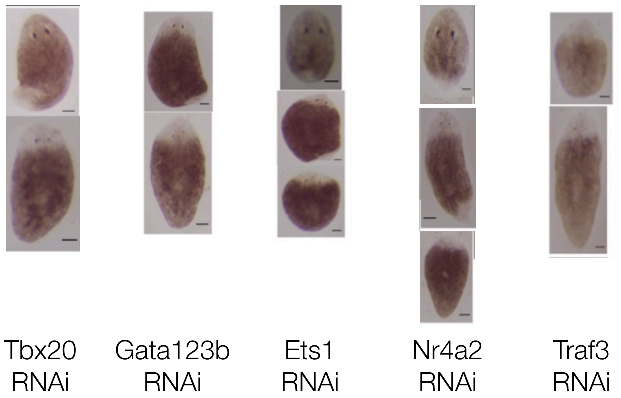

Supplemental figure 13. dsRNA-mediated RNA interference of the blastema TFs. (**A**) Efficiency of the RNAi after either 4 or 7 days from the first dsRNA injection, as for qPCR. Eight genes are shown, representative of the 20 that were knocked-down. For each gene, at each time point, both control (C, injected with GFP dsRNA) and target (T) genes are shown. The orange and red dashed lines represent 50% and 25% residual expression, respectively. (**B**) Five of the considered blastema TFs gave no macroscopic phenotype, neither at 7 dpa (shown), nor at 14 dpa (not shown).

…
