# Supplementary material for: Transcription Factors Active in the Anterior Blastema of Schmidtea mediterranea
Source: Biomolecules. 2021 Nov 28;11(12):1782. doi: 10.3390/biom11121782 (PMC8698962; doi:10.3390/biom11121782)
Supplement: Supplementary file 1 [file biomolecules-11-01782-s001.zip › FigureS2.pdf]

Supplemental figure 2

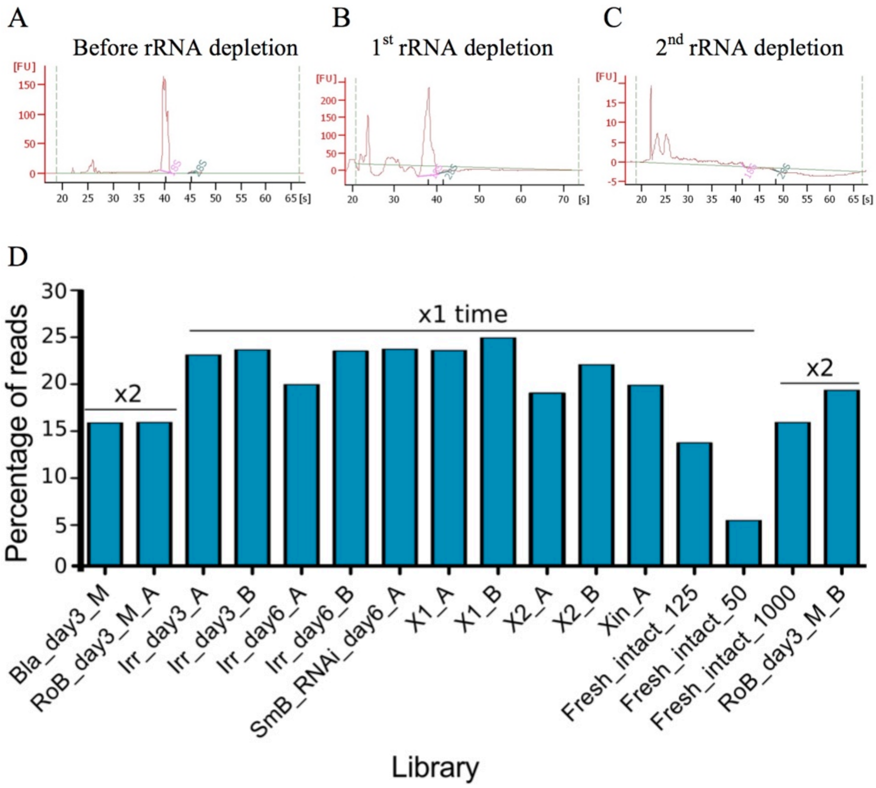

Supplemental figure 2. Effectivity of two rounds of rRNA depletion and distribution of reads mapping on rRNA seuences after RNA-seq. (**A-C**) Bioanalyzer plot of a representative sample before rRNA depletion (A) and after 1 (B) or 2 (C) rounds of rRNA depletion. The lack of the 28s peak owes to the fact that in planarian, as in other protostomes, mature 28s rRNA molecules are cleaved in two 28s rRNA subunits (28sα and 28sβ) [56]. Thus, during electrophoresis, the two subunits co-migrate with the 18s rRNA. (**D**) Reads that match rRNA sequences after 1 (1x) or 2 (2x) rounds of rRNA depletion.
