# Supplementary material for: Transcription Factors Active in the Anterior Blastema of Schmidtea mediterranea
Source: Biomolecules. 2021 Nov 28;11(12):1782. doi: 10.3390/biom11121782 (PMC8698962; doi:10.3390/biom11121782)
Supplement: Supplementary file 1 [file biomolecules-11-01782-s001.zip › FigureS3.pdf]

Supplemental figure 3

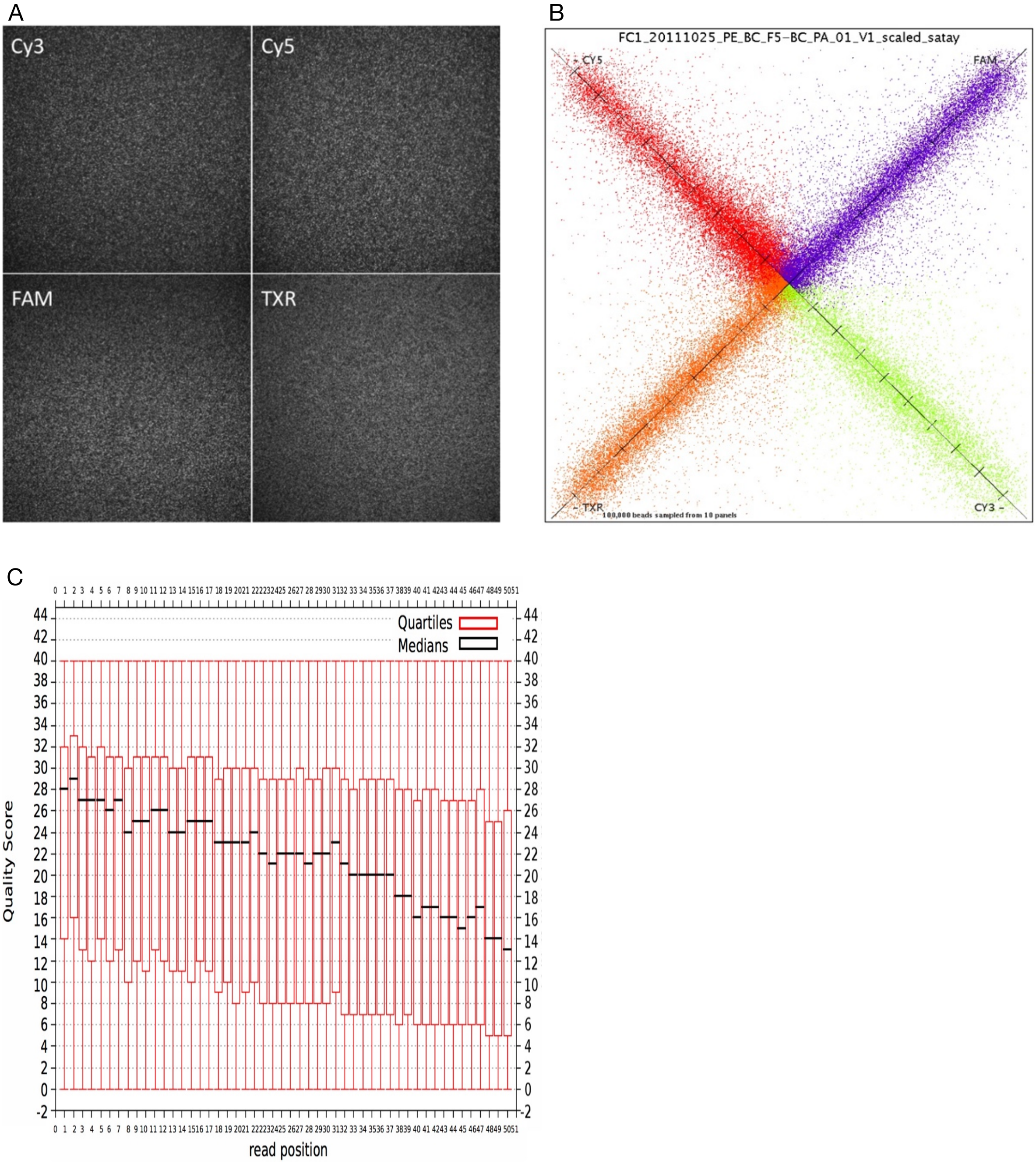

Supplemental figure 3. RNA-seq data quality check. (**A**) microscopic fluorescence images of one representative flow cell (FC1), for all the channels used (channel name written in each image). (**B**) The fluorescence data depicted in (A) represented in a satay plot. (**C**) Box-plot showing the scores of the RNA-seq data obtained from the FC1.

…
