# Supplementary material for: Transcription Factors Active in the Anterior Blastema of Schmidtea mediterranea
Source: Biomolecules. 2021 Nov 28;11(12):1782. doi: 10.3390/biom11121782 (PMC8698962; doi:10.3390/biom11121782)
Supplement: Supplementary file 1 [file biomolecules-11-01782-s001.zip › FigureS4.pdf]

Supplemental figure 4

A

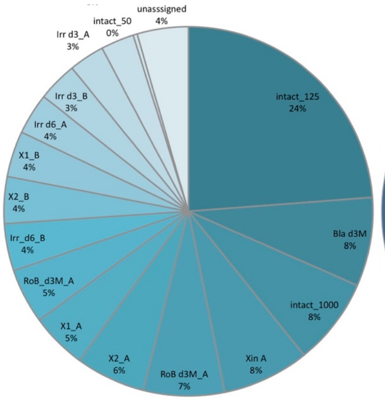

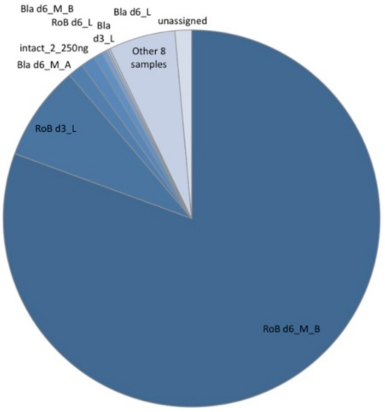

Sample	Reads number	%
intact_125	139391290	23.8
Bla d3M	45626513	7.8
intact_1000	44724829	7.6
Xin A	44636451	7.6
RoB d3M_A	41607130	7.1
X2_A	35769702	6.1
X1_A	28916762	4.9
RoB_d3M_A	28200512	4.8
Irr_d6_B	24228085	4.1
X2_B	24082308	4.1
X1_B	23490515	4.0
Irr d6_A	21240173	3.6
Irr d3_B	20329029	3.5
Irr d3_A	18237186	3.1
SmB_RNAi_d6	17020433	2.9
intact_50	2099852	0.4
unasssigned	26258468	4.5
Total	585859238	100.0

Sample	Reads Number	%
RoB d6_M_B	341732948	80.6
RoB d3_L	34673197	8.2
Bla d6_M_A	5713158	1.3
intact_2_250ng	5148841	1.2
Bla d6_M_B	3136913	0.7
RoB d6_L	1688341	0.4
Bla d3_L	816022	0.2
Bla d6_L	810964	0.2
Other 8 samples	24281675	5.7
unassigned	6023801	1.4
Total	424025860	100.0

B

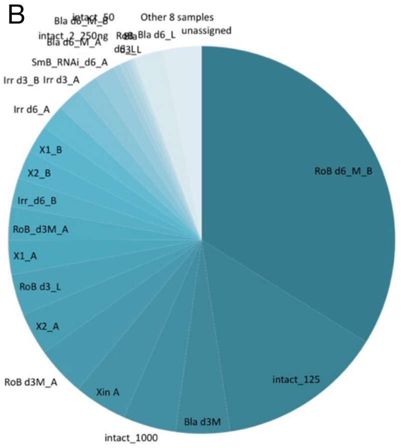

Sample	Reads number	%
RoB d6_M_B	341732948	33.8
intact_125	139391290	13.8
Bla d3M	45626513	4.5
intact_1000	44724829	4.4
Xin A	44636451	4.4
RoB d3M_A	41607130	4.1
X2_A	35769702	3.5
RoB d3_L	34673197	3.4
X1_A	28916762	2.9
RoB_d3M_A	28200512	2.8
Irr_d6_B	24228085	2.4
X2_B	24082308	2.4
X1_B	23490515	2.3
Irr d6_A	21240173	2.1
Irr d3_B	20329029	2.0
Irr d3_A	18237186	1.8
SmB_RNAi_d6_A	17020433	1.7
Bla d6_M_A	5713158	0.6
intact_2_250ng	5148841	0.5
Bla d6_M_B	3136913	0.3
intact_50	2099852	0.2
RoB d6_L	1688341	0.2
Bla d3_L	816022	0.1
Bla d6_L	810964	0.1
Other 8 samples	24281675	2.4
unassigned	32282269	3.2
Total	1009885098	100.0

C

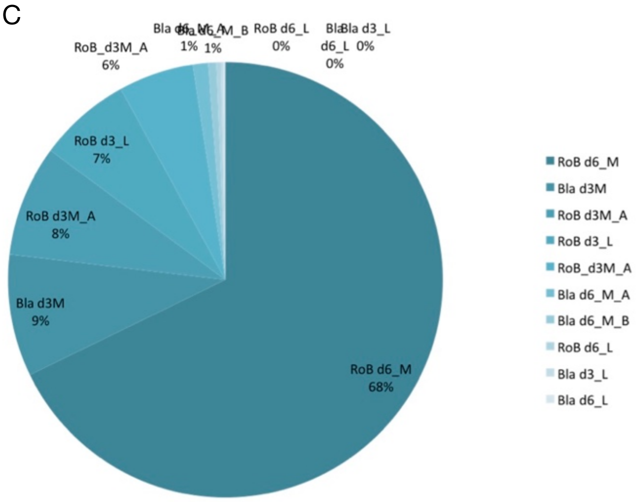

Sample	Reads number	%
RoB d6_M	341732948	67.8
Bla d3M	45626513	9.1
RoB d3M_A	41607130	8.3
RoB d3_L	34673197	6.9
RoB_d3M_A	28200512	5.6
Bla d6_M_A	5713158	1.1
Bla d6_M_B	3136913	0.6
RoB d6_L	1688341	0.3
Bla d3_L	816022	0.2
Bla d6_L	810964	0.2
Total	504005698	100.0

Supplemental figure 4. Sample-wise RNA-seq reads distribution in the two flow cells used in this study. (**A**) Percentage of assigned and unassigned reads distribution on FC1 (left) and FC2 (right). 95.52% and 98.6% of total reads were barcoded after sequencing in the two flow cells, respectively. (**B**) The data from FC1 and FC2 were merged. (**C**) Relative percentage of the total reads assigned to Blastema (Bla) and Rest of Body (RoB) samples, at both day3 and day6.
