# Supplementary material for: Transcription Factors Active in the Anterior Blastema of Schmidtea mediterranea
Source: Biomolecules. 2021 Nov 28;11(12):1782. doi: 10.3390/biom11121782 (PMC8698962; doi:10.3390/biom11121782)
Supplement: Supplementary file 1 [file biomolecules-11-01782-s001.zip › FigureS5.pdf]

Supplemental figure S5

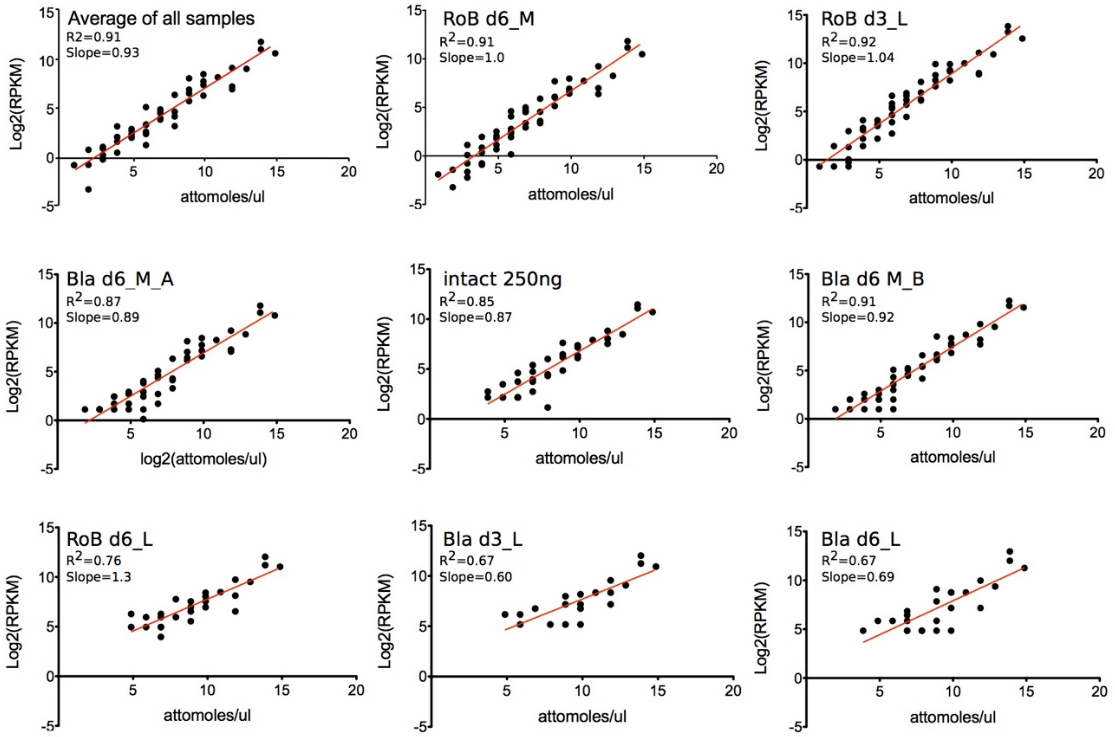

Supplemental figure S5. Analysis of the spike-in control added to the samples run in FC1. The slope, the calculated trendline and the concentration range covered by the Spike-in controls added to each sample run on FC1 is shown.

…
