# Supplementary material for: Transcription Factors Active in the Anterior Blastema of Schmidtea mediterranea
Source: Biomolecules. 2021 Nov 28;11(12):1782. doi: 10.3390/biom11121782 (PMC8698962; doi:10.3390/biom11121782)
Supplement: Supplementary file 1 [file biomolecules-11-01782-s001.zip › FigureS6.pdf]

Supplemental figure 6

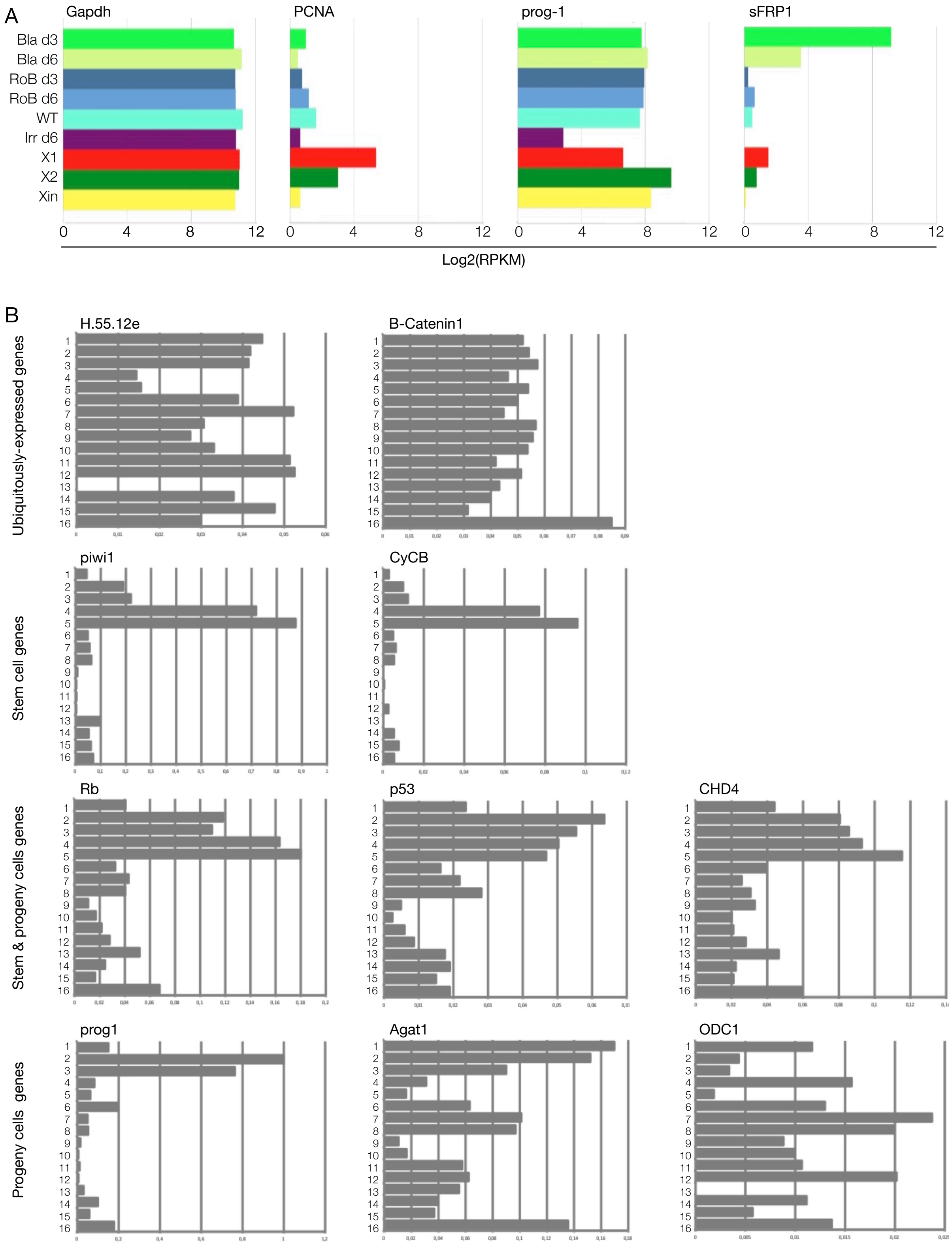

Supplemental figure 6. RNA-seq data relative to planarian genes for which a pattern of expression is known. (**A**) Expression data (as Log_2RPKM) of the ubiquitously expressed marker Gapdh, the stem cell-specific marker PCNA, the progeny marker prog-1 and the anterior marker sFRP1. The data are presented as the average of three biological replicates. (**B**) The expression data (as Log_2RPKM) of other representative genes is shown for individual samples from FC1. Samples are as follows: 1 = Xin_1; 2 = X2_2; 3 = X2_1; 4 = X1_2; 5 = X1_1; 6 = SmB_RNAi_1; 7 = RoB_d3_2; 8 = RoB_d6_1; 9 = Irr_d6_2; 10 = Irr_d6_1; 11 = Irr_d3_2; 12 = Irr_d3_1; 13 = WT_1; 14 = WT_2; 15 = WT_3; 16 = Bla_d3_1
