# Supplementary material for: Transcription Factors Active in the Anterior Blastema of Schmidtea mediterranea
Source: Biomolecules. 2021 Nov 28;11(12):1782. doi: 10.3390/biom11121782 (PMC8698962; doi:10.3390/biom11121782)
Supplement: Supplementary file 1 [file biomolecules-11-01782-s001.zip › FigureS7.pdf]

Supplemental figure 7

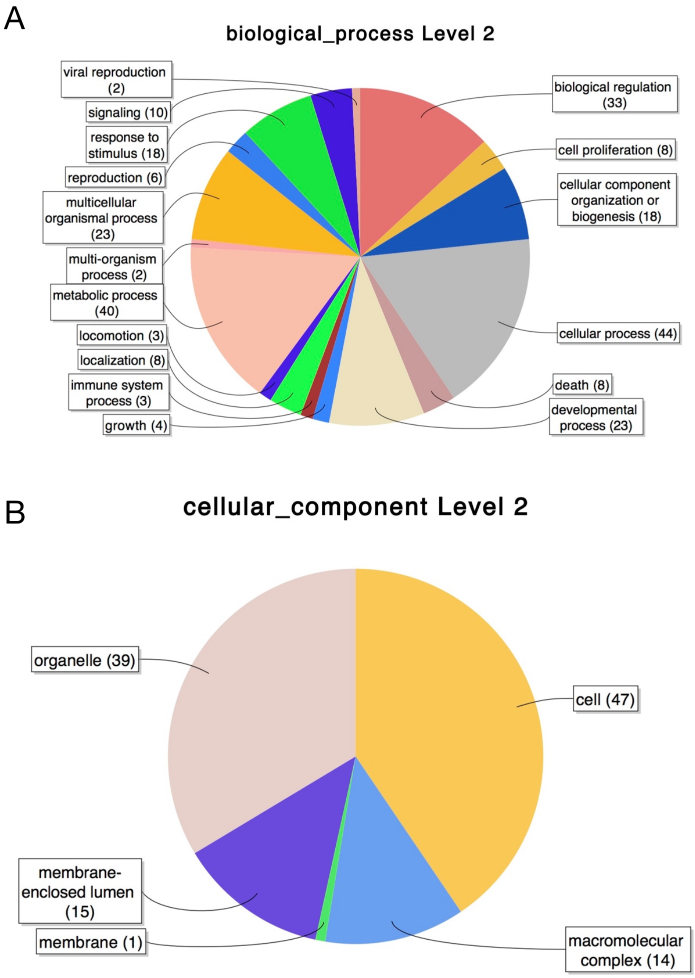

Supplemental figure 7. Additional gene ontology (GO) annotations. GO annotation of the shortlist of putative transcription factors according to the biological process in which they are involved (A) and the cellular components to which they were found associated (B).
