# Supplementary material for: Transcription Factors Active in the Anterior Blastema of Schmidtea mediterranea
Source: Biomolecules. 2021 Nov 28;11(12):1782. doi: 10.3390/biom11121782 (PMC8698962; doi:10.3390/biom11121782)
Supplement: Supplementary file 1 [file biomolecules-11-01782-s001.zip › FigureS8.pdf]

Supplemental figure 8

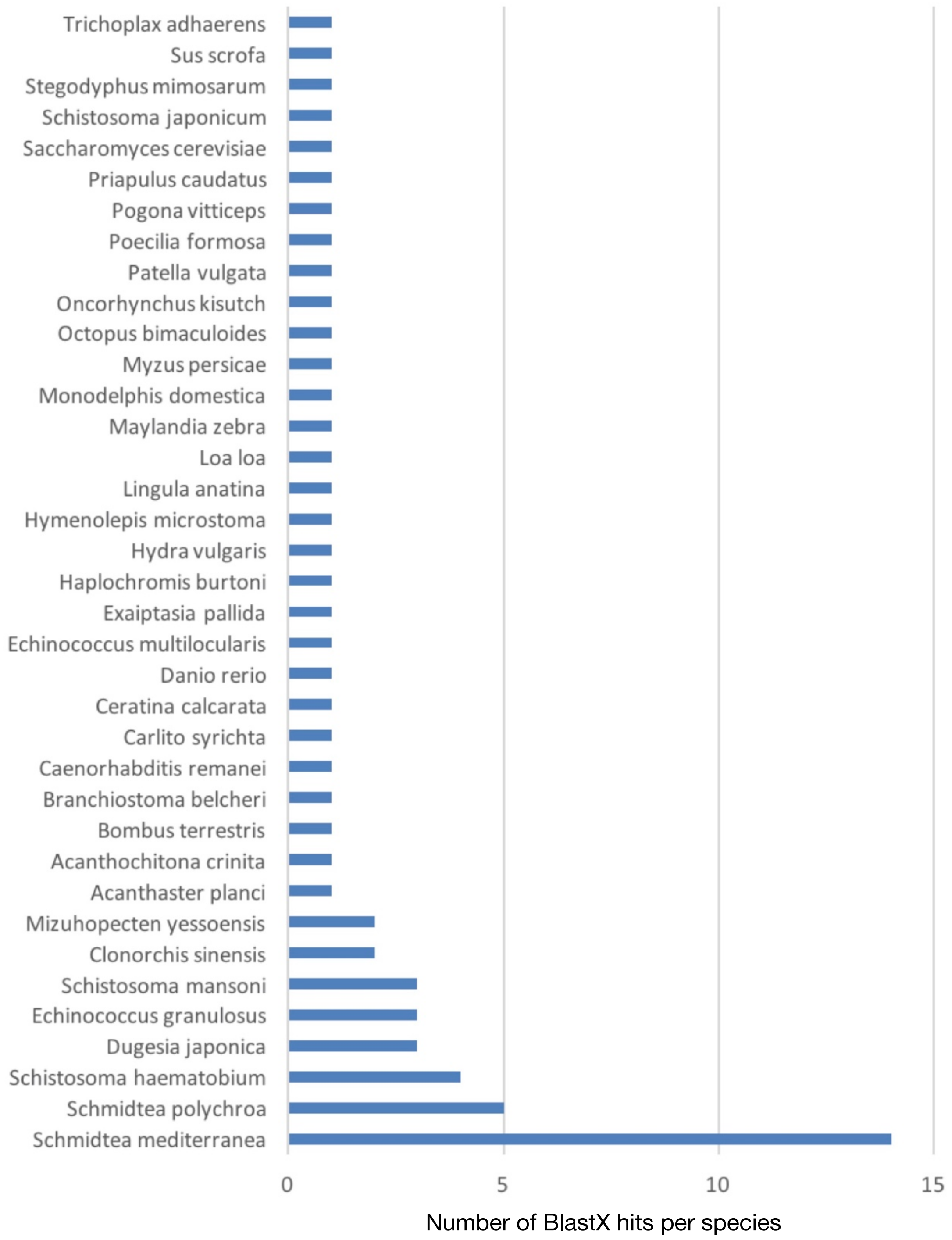

Supplemental figure 8. Species distribution of 65 candidates. The 65 putative TF genes were plotted according to the animal species that gave the highest score for the BLAST hit. A total of 22 putative TFs showed the highest homology to planarian genes while 32 showed the highest homology with plathyhelminthes gens.
