# Supplementary material for: Transcription Factors Active in the Anterior Blastema of Schmidtea mediterranea
Source: Biomolecules. 2021 Nov 28;11(12):1782. doi: 10.3390/biom11121782 (PMC8698962; doi:10.3390/biom11121782)
Supplement: Supplementary file 1 [file biomolecules-11-01782-s001.zip › FigureS9.pdf]

Supplemental figure 9

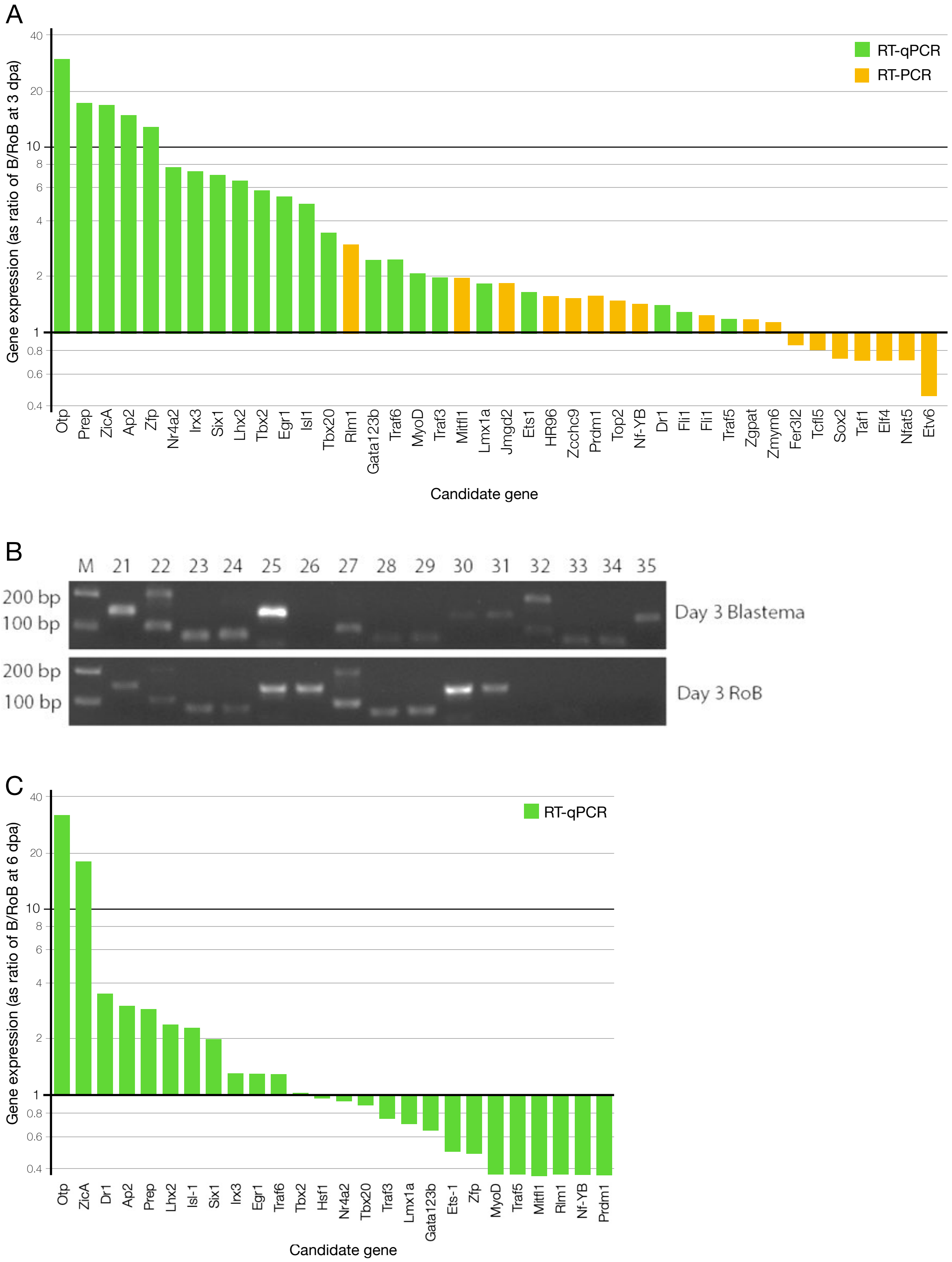

Supplemental figure 9. The expression of 40 transcription factor/transcriptional regulator genes found enriched in blastema samples. (**A**) Expression of 40 transcription factor/transcriptional regulator genes at 3 dpa, as for quantitative (green) or conventional RT-PCR (orange) quantification. Each bar represents the ratio between the expression of one gene in the Blastema and in the RoB samples. (**B**) Representative agarose gel electrophoresis of the products of conventional RT-PCR for 15 candidate genes used to quantify the gene expression after ethidium bromide stain. (**C**) Expression of 26 transcription factor/transcriptional regulator genes at 6 dpa, as for RT-qPCR. The gene expression is shown as the ratio between Blastema and RoB samples.
