# Supplementary material for: Transcription Factors Active in the Anterior Blastema of Schmidtea mediterranea
Source: Biomolecules. 2021 Nov 28;11(12):1782. doi: 10.3390/biom11121782 (PMC8698962; doi:10.3390/biom11121782)
Supplement: Supplementary file 1 [file biomolecules-11-01782-s001.zip › Table S1.pdf]

Table S1. Oligonucleotides used for RT-PCR and RT-qPCR.

RT-qPCR

Gene name	Primer and Probe	Primer sequence (5'-3')
Musashi	Forward	AGATTCCACGATGCCTGAAG
	Probe	56-FAM/CCGAAATC C/Zen/CCAGCCGGTGT/3IABkFQ
	Reverse	GCATCCACTAGTTGTCCGTTAG
Hsfl	Forward	TCGACACTGTTCAATGACTCG
	Probe	56-FAM/TAGATCAGC/Zen/AAGAACGGGTCAGGAAC/3IABkFQ
	Reverse	ATTTCCCAATCTACGTCCAGG
Tbx20	Forward	AGAAAACGGAGAATCAGGATGG
	Probe	56-FAM/AGGATCGGC/Zen/TTTACCAGCAACCA/3IABkFQ
	Reverse	CGGTACGCATATCACAGATCG
Gata123b	Forward	GCAATTTGAACAGATCGTACCG
	Probe	56-FAM/AGCCCAAAC/Zen/GAAGATTGTCAGCCT/3IABkFQ
	Reverse	CTTTGTAACGCTTGTGGACTG
Ap2	Forward	GATCGGAGCCATGTCAAATTC
	Probe	56-FAM/CGTTTCAGT/Zen/CTCAGTCAACCTCATCCAA/3IABkFQ
	Reverse	GGCATGACTACCGAAATCTACAC
Egr1	Forward	TGCTCGATTTGCTCCTATTCTG
	Probe	56-FAM/TGTCGAGTA/Zen/ATCATATCCCTCCGGCA/3IABkFQ
	Reverse	ACTATATCCCCTTTCCGTTTGTG
Zfp	Forward	CGTCACCGTCATTGAAAAGTG
	Probe	56-FAM/ACAAAGTCA/Zen/AACAGTCGGCCTCACC/3IABkFQ
	Reverse	GGTTAGATGGTGTCGGGATTG
Myod	Forward	CCCAGAAAATTCGAGAACAGC
	Probe	56-FAM/AATCGCCGG/Zen/AAAACAAAAGCCACT/3IABkFQ
	Reverse	CTGATCAATGTCGGAAGCAAC
	Forward	AACTCCATTTCTAACCCCACG

Zfp	Probe	56-FAM/ACGAGAGTC/Zen/GGGAAAACTTAGCAAGC/3IABkFQ
	Reverse	GCCGTTTACAATATGAGCAGG
Dr1	Forward	TTGGAAGCGAGGGAACTAATG
	Probe	56-FAM/TTGCAAGCT/Zen/AGATTCGCCTGTCCA/3IABkFQ
	Reverse	CCAAAGCTGTTAGAACGTGATC
Lmx1a	Forward	AGACCTGAACCACACGAAC
	Probe	56-FAM/AGAAAATCT/Zen/CGCCTCGGAAACTGGAC/3IABkFQ
	Reverse	TCCTAAGCCATCGAGAAAGTTG
Smarcb1	Forward	GAATTCCCAAATTGTCGCGG
	Probe	56-FAM/CGTCTTGGG/Zen/ATTGGCTGGATTGGA/3IABkFQ
	Reverse	GCTTGTATATTGGCGCATTTGG
Ets-1	Forward	ATGGGTAAACGTGTCAGTCG
	Probe	56-FAM/CGGACAGCG/Zen/ATTTTCTCTCTGGCA/3IABkFQ
	Reverse	CTGTGTTCTTTGTATCGGCTTG
Isl-1	Forward	TCTGTTCCTTGCTCCGTTTG
	Probe	56-FAM/CCGGCAATG/Zen/TGAAATTGAGTGGCA/3IABkFQ
	Reverse	GGATGGAAGCAGTGTTTGTG
Nr4a2	Forward	CAACTGTATAAACCGATTGAGCAC
	Probe	56-FAM/AAGAGAGTC/Zen/TGAAGGGTCGACGAGG/3IABkFQ
	Reverse	AAATGTATCAAAGTGGGAATGGTG
Lhx2	Forward	GCAACAGACCTTTCAATCCG
	Probe	56-FAM/TGGTCGAAT/Zen/GCTGAATCCGGGAG/3IABkFQ
	Reverse	TGAGTTGGTAATGCGGGTG
Traf6	Forward	GACATCCACCCATCCACATAC
	Probe	56-FAM/ACTTTTCAC/Zen/TAAGCGTTCCCCACGG/3IABkFQ
	Reverse	ATGCTATTGCTTCGGAGTCTC
Traf5	Forward	AGAAGTTTTGTCCGGGTGAC
	Probe	56-FAM/CGCAATTCG/Zen/ATGATTGGTCGGTTGA/3IABkFQ

	Reverse	CATCTCCACAGGTTTCCGG
Traf3	Forward	GGTTACGTCGATTTTGCATGG
	Probe	56-FAM/CCTTTGCGT/Zen/TATTCCGAATGGACTCGTA/3IABkFQ
	Reverse	CAGAAAACGGCTACCAAATGTG
Tbx2	Forward	CTCAATTCATAGGCGTAACAGC
	Probe	/56-FAM/CAAAAGGAT/ZEN/TCCGTGATACAGGTGGAGG/3IABkFQ/
	Reverse	AAGTCGAAATTGCAGAACCATTC
Smc2	Forward	TTTCCGCTTCTCATCTTCCAG
	Probe	CCGCCAGACTGATTGAAATGTTTGCT
	Reverse	AGATCGGGAAATTGCTGTCG
Tigd1	Forward	TGCAGTCCTAGTAACCAAAGC
	Probe	TGCCGATCCAGTTTCACCTTGAACTT
	Reverse	AACCCTTGCAGCTTCTACG
H2a	Forward	GTTGGCCGAATTCACAGAATG
	Probe	CAGAGCGAGTTGGTGCTGGTG
	Reverse	TGTTTTCTTGTTATCGCGTGC
Irx3	Forward	CTTGTAAAGGTTTGAGGCGATG
	Probe	ACCGAATGTTGGAGCTGGAGGTT
	Reverse	ATTCCACAGATACCAGCACAG
Rlm1	Forward	GATCCGAGAATTTATACACAAGGTC
	Probe	AAGGCCAGCGAACTGAATCGACT
	Reverse	TGACCGTAAAAGAGCTAGCAC
Tufm	Forward	TCCTTAGTTTGAGGCATAGCG
	Probe	CTGCAGCAACAACTAAAATAGCCCCATC
	Reverse	TGCACATACAGATTGTCCTGG
Mitfl1	Forward	CCCTTTCATGTCCTGTAACCG
	Probe	TTGCATGATTCATACTGACCTCTGACCG
	Reverse	TTGATATTGACCCTGTACTGACG

Nf-yb	Forward	TTCTACTCACATCCAAAGCGG
	Probe	TGAGCTACCTAAACCACATGCAGCC
	Reverse	TCCTTCACCTATCCTTTGTCTTG
Prep	Forward	GCCACTCAGACTAATTTGACTTTG
	Probe	CCGACGCAGAATCCTACAACCGATG
	Reverse	TGGACTTATCTTTGCCTGGTG
Prdm1	Forward	AGATCAGAGCCAGGACAAATG
	Probe	CGTCAGCGGACTCAAATATCATTGGACA
	Reverse	ACATCACACTAGAGAAACACCG
Six1	Forward	CGGAGTTGGATAAGGATTTTGAC
	Probe	AACGATTTGGGATGGAGATGAAACGAGT
	Reverse	GGGTGCTGTGGGTAAATATAGAG
Zica	Forward	GTCACAGCCTTCAAAATCGC
	Probe	TCACTACGAGCAAACAGTTTCCCACA
	Reverse	TCACATTCGAGTTCACACTGG
Otp	Forward	GAGGTGGCATAGCAGTCATAC
	Probe	ATATCTTTGGGACCAGGAAGCGGC
	Reverse	CCAGCACATTTTCAAGAACCG
Smed-Adh-3	Forward	CAGATGCGTATACACTCAGTGGTTG
	Probe	CCACTATCCCAGAACCTTCATGTC
	Reverse	AGGAGTTTTTCCTGTTGTCCT
Smed-AGAT-1	Forward	GGTTGGAAGATTGTGAAGGG
	Probe	TGTATGAAGGCATGAGTTACAAGTGGC
	Reverse	CCAACCTCTCGCTTTTCA
Smed-bruli-1 (Celf)	Forward	AACACTCCCGCATTAGGCTTATC
	Probe	ACCCTTCAGGACCAGTTACAATATTG
	Reverse	ATACCACTGCTTTGTCTCTA
Smed-CyclinB (Ccnb1)	Forward	GCGATGTCCTTGGTCGACAT
	Probe	TCTCGTGAAATTAGCCCTAGG

	Reverse	AAGTTTCGGCGCATAATGAGAA
Smed-GAPDH	Forward	GCTGTCGGTCAAGTCATT
	Probe	AAACGGAAAACTCACCGGAATGGC
	Reverse	GGATACATCGGCTACAGGTA
Smed-myhc-1 (T-mus)	Forward	TGAAGAGCGAGCTGATCAAGC
	Probe	AGCTCGGTATCTGTTAGTC
	Reverse	GCGGATTGATGTCGCAGTTATAG
Smed-NB32.1g	Forward	GTTCTCGCTGTGTTATTTGTTTACGT
	Probe	TGTCGAGTCGCATTTTAAATCGGCG
	Reverse	GGCACTCATTTCTCGTTTCTGTATT
Smed-piwi-1	Forward	AGTTCCTGTTCCAACGCATTATG
	Probe	CTGAACTCGTTGGCAAGA
	Reverse	CTGGAGGAGTAACACCACGATGA
Smed-PCNA	Forward	GTGATGGTTTTGAGACTTATCGATG
	Probe	TGTTAGGGAATCATTACTACCAAGCGCC
	Reverse	GTTTCACTTGAATCAGCGGC

RT-PCR primers

Gene name	Primer	Primer sequence (5'-3')	Product size (bps)
Smc2	Forward	TTTCCGCTTCTCATCTTCCAG	146
	Reverse	AGATCGGGAAATTGCTGTCG	
Top2	Forward	CACGCTGGCACCAAATTAAG	147
	Reverse	TTCCCCAAAACATCGAGACC	
Fli1	Forward	CTTGGACTATCGGAAATTTGTGG	148
	Reverse	CGGCCATTGTGACTTGATATTG	
Rfc3	Forward	TGCTTTCTGTCCGAGATTCATC	149
	Reverse	GACTTGCCATTGAAACTGACG	

Tigd1	Forward<br>Reverse	TGCAGTCCTAGTAACCAAAGC<br>AACCCTTGCAGCTTCTACG	150
Etv6	Forward<br>Reverse	TCATCTTTGTCCACCCAGC<br>CCAAGATTGCATTTGCTCCAG	151
Tcf15	Forward<br>Reverse	ACCTTCATACGCTTCTCATTACC<br>CAGAAACGGCGATTGGATTTG	152
Taf11	Forward<br>Reverse	GAAGCCTTAGATTATCGGGACAG<br>CGCCTAACCGATTCTTTGATATG	153
Elf4	Forward<br>Reverse	GGCAAATTCTCCTAACGATGTTG<br>TCTCCCCAAATTATCGACACG	154
Yeats4	Forward<br>Reverse	GGTAATACTGCGAGGTATCTTCAG<br>CATTATATGGTTGGAAAAATATGCACC	155
Pcbp3	Forward<br>Reverse	GAAACCGCACGAATCTCATTG<br>CAACGACTAATCATTTTACCCAGC	156
Zgpat	Forward<br>Reverse	CCGCTTCGTTAATTTCCATAGG<br>GCAGTTTTGTAGTGGAACTCG	157
Rnf11	Forward<br>Reverse	GCCGTGATATTCTTTGAGGACC<br>CCATTAAATCAAGAATCCGGCG	158
Zcchc9	Forward<br>Reverse	TTCTCTTCGGCAGTACACTTG<br>TCGTTGGTGTTTCAGTTGTAGAG	159
Jmjd2	Forward<br>Reverse	TCTACGGTGCCAATGTTAGC<br>TGGTTCTTTCAAAACATCGCTG	160
H2a	Forward<br>Reverse	GTTGGCCGAATTCACAGAATG<br>TGTTTTCTTGTTATCGCGTGC	161
Brd3	Forward<br>Reverse	TGGATAATCCGGTAATTGATAGGC<br>CCTTTTGTTATGAGATACGAAGCTG	162
Irx3	Forward<br>Reverse	CTTGTAAAGGTTTGAGGCGATG<br>ATTCCACAGATACCAGCACAG	163

Zmym6	Forward<br>Reverse	AATGAGATCGGGGCATGAAG<br>ATTCAACTTCATTTCGTAATTCAAAGAG	164
Ep300	Forward<br>Reverse	TCCTTTTCTCGTTTCTCCTGG<br>GCAATTGATAATCCACGCTCAG	165
Rlm1	Forward<br>Reverse	GATCCGAGAATTTATACACAAGGTC<br>TGACCGTAAAAGAGCTAGCAC	166
Tufm	Forward<br>Reverse	TCCTTAGTTTGAGGCATAGCG<br>TGCACATACAGATTGTCCTGG	167
Dsp1	Forward<br>Reverse	ACGAATTTGTTACATTTCTACCGG<br>GAAAGTCAAATTGCTGCTGAGG	168
Hsf	Forward<br>Reverse	ATGACAGTTACCAAGCACCA<br>ATTTCAGTTCGGCGCATTTG	169
Mitfl1	Forward<br>Reverse	CCCTTTCATGTCCTGTAACCG<br>TTGATATTGACCCTGTACTGACG	170
Nf-yb	Forward<br>Reverse	TTCTACTCACATCCAAAGCGG<br>TCCTTCACCTATCCTTTGTCTTG	171
Sox2	Forward<br>Reverse	AGAGCCAACCATCAGAAAGAG<br>TCCCGTGTTTATCTTGAGGC	172
Prep	Forward<br>Reverse	GCCACTCAGACTAATTTGACTTTG<br>TGGACTTATCTTTGCCTGGTG	173
Nfat5	Forward<br>Reverse	CTCGGTTAGTGACAGTGAAGG<br>TTCGGGCTGGTTAATGAGAAG	174
Prdm1	Forward<br>Reverse	AGATCAGAGCCAGGACAAATG<br>ACATCACACTAGAGAAACACCG	175
Zfp	Forward<br>Reverse	TCTCGGCGTAAAGTTCATCC<br>CTCTCGTTTGCAATCATTGAGG	176
Six1	Forward<br>Reverse	CGGAGTTGGATAAGGATTTTGAC<br>GGGTGCTGTGGGTAAATATAGAG	177

Hr96	Forward	GTTCCATTCAAAACAGTCGGC	178
	Reverse	TCAAACTTTCTCAGCCCTACG	
Fer3l-2	Forward	TTGTTCATCCTTCTCCGTTCTC	130
	Reverse	GTTTCATGGCGGGTCAATTG	
Zica	Forward	GTCACAGCCTTCAAAATCGC	148
	Reverse	TCACATTCGAGTTCACACTGG	
Otp	Forward	GAGGTGGCATAGCAGTCATAC	148
	Reverse	CCAGCACATTTTCAAGAACCG	
