# Supplementary material for: Transcription Factors Active in the Anterior Blastema of Schmidtea mediterranea
Source: Biomolecules. 2021 Nov 28;11(12):1782. doi: 10.3390/biom11121782 (PMC8698962; doi:10.3390/biom11121782)
Supplement: Supplementary file 1 [file biomolecules-11-01782-s001.zip › Table S2.pdf]

Table S2. Oligonucleotides used for the synthesis of the riboprobes.

ISH and dsRNA

Name	Gene name	Primer sequence (5'-3')	Product size (bps)
Cand1_ISH_Sp6_F<br>Cand1_ISH_T7_R	Musashi	ATTTAGGTGACACTATAGAGATTCCACGATGCCTGAAG<br>TAATACGACTCACTATAGGGGCACGATTCAACCTTTCCAT	753
Cand2_ISH_Sp6_F<br>Cand2_ISH_T7_R	Hsfl	ATTTAGGTGACACTATAGCAACCGGAGGAAATAACCAA<br>TAATACGACTCACTATAGGGCAACGTTAGGCGACAATGTG	827
Cand3_ISH_Sp6_F<br>Cand3_ISH_T7_R	Tbx20	ATTTAGGTGACACTATAGCCGGAGGACAAATTCGTAGA<br>TAATACGACTCACTATAGGGAATTCCGACGACAACGATTC	793
Cand4_ISH_Sp6_F<br>Cand4_ISH_T7_R	Gata123b	ATTTAGGTGACACTATAGTGATCGTGTTGGAAATGGAA<br>TAATACGACTCACTATAGGGCGCGAATGTGTTAATTGTGG	769
Cand5_ISH_Sp6_F<br>Cand5_ISH_T7_R	Ap2	ATTTAGGTGACACTATAGCTGAATTTGGGAGACCGTGT<br>TAATACGACTCACTATAGGGACGAGCCAATCTCACAGCTT	998
Cand6_ISH_Sp6_F<br>Cand6_ISH_T7_R	Egr1	ATTTAGGTGACACTATAGTACGTGGCAAAACGCAATAA<br>TAATACGACTCACTATAGGGAGCAAACGTTCGAGGAGGTA	729
Cand7_ISH_Sp6_F<br>Cand7_ISH_T7_R	Zfp	ATTTAGGTGACACTATAGAAAGCTCGCAAAACCGATAA<br>TAATACGACTCACTATAGGGATTCCAGTTGCTACCCAACG	972
Cand8_ISH_Sp6_F<br>Cand8_ISH_T7_R	Myod	ATTTAGGTGACACTATAGCAGGTTCCACTTGTCCGATT<br>TAATACGACTCACTATAGGGCAAGCATTGACCATGTGACC	833
Cand9_ISH_Sp6_F<br>Cand9_ISH_T7_R	Zfp	ATTTAGGTGACACTATAGGCACACAGTTGCCGTTTAGA<br>TAATACGACTCACTATAGGGAGCCTTTCTCAACCGTCTCA	924
Cand10_ISH_Sp6_F<br>Cand10_ISH_T7_R	Dr1	ATTTAGGTGACACTATAGTGGATCCAGTTGATTTAATTGC<br>TAATACGACTCACTATAGGGTTGATTATTGGCACCGTCAG	500
Cand11_ISH_Sp6_F<br>Cand11_ISH_T7_R	Lmx1a	ATTTAGGTGACACTATAGAGACCTGAACCACACGAACC<br>TAATACGACTCACTATAGGGTACCGATGCCATTCTGTGAA	765
Cand12_ISH_Sp6_F<br>Cand12_ISH_T7_R	Smarcb1	ATTTAGGTGACACTATAGTCCAAATGCGCCAATATACA<br>TAATACGACTCACTATAGGGTTGGTCAGATCGGCAATACA	819

Cand13_ISH_Sp6_F<br>Cand13_ISH_T7_R	Ets-1	ATTTAGGTGACACTATAGAATGTCGTCGGATCTCCTTG<br>TAATACGACTCACTATAGGGTCTTTGTACTGGCCCCAAAG	828
Cand14_ISH_Sp6_F<br>Cand14_ISH_T7_R	Isl-1	ATTTAGGTGACACTATAGTCGATCTGGAAATGTCACCA<br>TAATACGACTCACTATAGGGATTTGTTTGCACGACCATCA	886
Cand15_ISH_Sp6_F<br>Cand15_ISH_T7_R	Nr4a2	ATTTAGGTGACACTATAGTGTGACAATTCCGGTGAAAA<br>TAATACGACTCACTATAGGGCTCGAATGCGATGATCAAGA	965
Cand16_ISH_Sp6_F<br>Cand16_ISH_T7_R	Lhx2	ATTTAGGTGACACTATAGTGCCTGATGAATCATTTCCA<br>TAATACGACTCACTATAGGGTGTCAATTGTTTGCGGTGTT	839
Cand17_ISH_Sp6_F<br>Cand17_ISH_T7_R	Traf6	ATTTAGGTGACACTATAGATGGAATTGCGAAGACCAAG<br>TAATACGACTCACTATAGGGTTGCAATCACGTTCTGAAGC	910
Cand18_ISH_Sp6_F<br>Cand18_ISH_T7_R	Traf5	ATTTAGGTGACACTATAGCGGAAACCTGTGGAGATGAT<br>TAATACGACTCACTATAGGGTGGAATTTGGAAGCATGTCA	870
Cand19_ISH_Sp6_F<br>Cand19_ISH_T7_R	Traf3	ATTTAGGTGACACTATAGGGAAACCCAAAAGAGGCATT<br>TAATACGACTCACTATAGGGCCTCATTGAAAGCCTGGAAA	738
Cand20_ISH_Sp6_F<br>Cand20_ISH_T7_R	Tbx2	ATTTAGGTGACACTATAGGTGGGAGCAATTTCATGCTT<br>TAATACGACTCACTATAGGGCGATGAATCAGGGGTCAGAT	856
cand21_tr5_Sp6_F<br>cand21_tr5_T7_R	Smc2	ATTTAGGTGACACTATAGTTCACACGCAATCTTTGCTC<br>TAATACGACTCACTATAGGGCGTCATGTCGTTTACGGTTG	876
cand22_tr5_Sp6_F<br>cand22_tr5_T7_R	Top2	ATTTAGGTGACACTATAGTCGTCCAAGTTCTTGTGCAG<br>TAATACGACTCACTATAGGGGTGTTGCCTTCACCCAGTTT	801
cand23_tr5_Sp6_F<br>cand23_tr5_T7_R	Fli1	ATTTAGGTGACACTATAGTGGAGCAGCAAACAAACAAG<br>TAATACGACTCACTATAGGGGCTGCTGCGTGTTGAAATAA	831
cand24_tr5_Sp6_F<br>cand24_tr5_T7_R	Rfc3	ATTTAGGTGACACTATAGCCAAGCTAACCGGATTCAAA<br>TAATACGACTCACTATAGGGAAAGTCGTCCCCTCACTTCA	773
cand25_tr5_Sp6_F<br>cand25_tr5_T7_R	Tigd1	ATTTAGGTGACACTATAGAACCAACGCTAAAGCGAAAA<br>TAATACGACTCACTATAGGGTTTTCAACGCTCGTGGATTT	744
cand26_tr5_Sp6_F<br>cand26_tr5_T7_R	Etv6	ATTTAGGTGACACTATAGGCCCAGAAATCGTTGTTTGT<br>TAATACGACTCACTATAGGGGGAGGAGGAGGTGATGATGA	894

cand27_tr5_Sp6_F<br>cand27_tr5_T7_R	Tcf15	ATTTAGGTGACACTATAGGCAAAAGAACGCGAAAGGT<br>TAATACGACTCACTATAGGGCAACAATACAAATCGGCAAGG	500
cand28_tr5_Sp6_F<br>cand28_tr5_T7_R	Taf11	ATTTAGGTGACACTATAGGCTTAACATCGTTTCAAATTTAGG<br>TAATACGACTCACTATAGGGTCGCCTAACCGATTCTTTGA	564
cand29_tr5_Sp6_F<br>cand29_tr5_T7_R	Elf4	ATTTAGGTGACACTATAGAACCATTGAGGTTTGGCAAG<br>TAATACGACTCACTATAGGGACATCACGTTGGACACCAAA	778
cand30_tr5_Sp6_F<br>cand30_tr5_T7_R	Yeats4	ATTTAGGTGACACTATAGAATTTTGTTTTAAAATTTCGGTTTG<br>TAATACGACTCACTATAGGGATTCAACAAATCATTCCATATCTTCC	700
cand31_tr5_Sp6_F<br>cand31_tr5_T7_R	Pcbp3	ATTTAGGTGACACTATAGTCATCCGATTGCTCTGACTG<br>TAATACGACTCACTATAGGGTAATGGCAATTGCTGATCCA	704
cand32_tr5_Sp6_F<br>cand32_tr5_T7_R	Zgpat	ATTTAGGTGACACTATAGCCATCCGGAAATTAGACCAA<br>TAATACGACTCACTATAGGGGATCGGATTGACACGACCTT	821
cand33_tr5_Sp6_F<br>cand33_tr5_T7_R	Rnf11	ATTTAGGTGACACTATAGCAACACCGGGAGTATCTCGT<br>TAATACGACTCACTATAGGGTCAAGAGCGAAACAAACGAA	710
cand34_tr5_Sp6_F<br>cand34_tr5_T7_R	Zcchc9	ATTTAGGTGACACTATAGTCTGGAAAGTTTAAGAAAAACGAA<br>TAATACGACTCACTATAGGGTCTCCGCCAACAACTTTTAGA	590
cand35_tr5_Sp6_F<br>cand35_tr5_T7_R	Jmjd2	ATTTAGGTGACACTATAGCCCATTAGCCAGAGCACATT<br>TAATACGACTCACTATAGGGATGGATGAGGCGTAGAATCG	811
cand36_tr5_Sp6_F<br>cand36_tr5_T7_R	H2a	ATTTAGGTGACACTATAGTTATGTCTGGTCGTGGCAAA<br>TAATACGACTCACTATAGGGTCATTTGAAGTCTTTTTCGGTAA	370
cand37_tr5_Sp6_F<br>cand37_tr5_T7_R	Brd3	ATTTAGGTGACACTATAGCCTGTTGATCACGTTGCCTA<br>TAATACGACTCACTATAGGGCAATTCTTGAACGGGCAACT	862
cand38_tr5_Sp6_F<br>cand38_tr5_T7_R	Irx3	ATTTAGGTGACACTATAGCGGAGGAATTCCAGATCAGA<br>TAATACGACTCACTATAGGGTTTCTTTGCACTTCGTGTCG	719
cand39_tr5_Sp6_F<br>cand39_tr5_T7_R	Zmym6	ATTTAGGTGACACTATAGAATCGCAAAATGGAAGAAGC<br>TAATACGACTCACTATAGGGCCCTTTCGTGCATCATCTTT	992
cand40_tr5_Sp6_F<br>cand40_tr5_T7_R	Ep300	ATTTAGGTGACACTATAGTCATCATTCCGTCTGATCCA<br>TAATACGACTCACTATAGGGATTGGTGGCTTTCGACTGAC	940

cand41_tr5_Sp6_F<br>cand41_tr5_T7_R	Rlm1	ATTTAGGTGACACTATAGAACGGAGAATCGGGCTATTT<br>TAATACGACTCACTATAGGGCGATCGTCTTCCTCGTGTAAA	829
cand42_tr5_Sp6_F<br>cand42_tr5_T7_R	Tufm	ATTTAGGTGACACTATAGGGTCATGTTGATCATGGGAAA<br>TAATACGACTCACTATAGGGAGATCGACCACCCTCATTTG	909
cand43_tr5_Sp6_F<br>cand43_tr5_T7_R	Dsp1	ATTTAGGTGACACTATAGTGGCAGTCCATGCAATTAAA<br>TAATACGACTCACTATAGGGTCTCGATGGAATGGCAGTTT	593
cand44_tr5_Sp6_F<br>cand44_tr5_T7_R	Hsf	ATTTAGGTGACACTATAGTTGGAATCAAGACGGGAAAG<br>TAATACGACTCACTATAGGGTAGTCCTGTGGTGGTGCTTG	777
cand45_tr5_Sp6_F<br>cand45_tr5_T7_R	Mitfl1	ATTTAGGTGACACTATAGACCAGCAAAAGCCTTCTTCA<br>TAATACGACTCACTATAGGGATTTCCACCGGAAGAATGTG	729
cand46_tr5_Sp6_F<br>cand46_tr5_T7_R	Nf-yb	ATTTAGGTGACACTATAGCCAACCGTTCCAGAAAACAT<br>TAATACGACTCACTATAGGGTGTTGCCCAGGTCAATGATA	902
cand47_tr5_Sp6_F<br>cand47_tr5_T7_R	Sox2	ATTTAGGTGACACTATAGGCAACGTCAACAAAACATGG<br>TAATACGACTCACTATAGGGTTGAGATTGGTTGCAATGGA	821
cand48_tr5_Sp6_F<br>cand48_tr5_T7_R	Prep	ATTTAGGTGACACTATAGGTGAACAAGCAACTGCCTCA<br>TAATACGACTCACTATAGGGCCTGGTGAGCCAGAAGAGTC	829
cand49_tr5_Sp6_F<br>cand49_tr5_T7_R	Nfat5	ATTTAGGTGACACTATAGAAAATTCCACGACTGCCATC<br>TAATACGACTCACTATAGGGCATCACAATAGGGCGGAACT	760
cand50_tr5_Sp6_F<br>cand50_tr5_T7_R	Prdm1	ATTTAGGTGACACTATAGTATGACGCAAGCCAATTTCA<br>TAATACGACTCACTATAGGGCCAATTGCGTGAAACCTTTT	740
cand51_tr5_Sp6_F<br>cand51_tr5_T7_R	Zfp	ATTTAGGTGACACTATAGATCCCCAAAAGTGACAGACG<br>TAATACGACTCACTATAGGGCCGGTGAATTGCTTCTTGAT	899
cand52_tr5_Sp6_F<br>cand52_tr5_T7_R	Six1	ATTTAGGTGACACTATAGTGGAACCGAATCATCAAACA<br>TAATACGACTCACTATAGGGAAAAGCCAGGAATTGTGGTG	779
cand53_tr5_Sp6_F<br>cand53_tr5_T7_R	Hr96	ATTTAGGTGACACTATAGTCTTCCCCATTGAAATGCTC<br>TAATACGACTCACTATAGGGGATTGACGGCTTTGTCGAAT	792
cand54_tr5_Sp6_F<br>cand54_tr5_T7_R	Fer3l-2	ATTTAGGTGACACTATAGTCCGATTTGAATCAATCTCTCA<br>TAATACGACTCACTATAGGGTTGGTTTGATTTCAGGAGCA	478

cand55_tr5_Sp6_F<br>cand55_tr5_T7_R	Zica	ATTTAGGTGACACTATAGCGAACTATGGCAGACAAGCA<br>TAATACGACTCACTATAGGGGTCCGTTTTGTGAGGCATTT	903
cand56_tr5_Sp6_F<br>cand56_tr5_T7_R	Otp	ATTTAGGTGACACTATAGCTTGCAAAGCAAAAACGACA<br>TAATACGACTCACTATAGGGGAACGCTTGATTTTGCCATT	938
cand57_tr5_Sp6_F<br>cand57_tr5_T7_R	Top2a	ATTTAGGTGACACTATAGGGCACTGGATGGAAAACATT<br>TAATACGACTCACTATAGGGACTTCGAACGGGCATATTCA	792
cand58_tr5_Sp6_F<br>cand58_tr5_T7_R	Foxf	ATTTAGGTGACACTATAGGGCAGACCTGGTAAAGGACA<br>TAATACGACTCACTATAGGGGCAACAGCTGCTACAGTGGA	704
cand59_tr5_Sp6_F<br>cand59_tr5_T7_R	Pax2/5/8	ATTTAGGTGACACTATAGTGATCCGTGCCATTCTAATTC<br>TAATACGACTCACTATAGGGAACGACTTTAGAAACTGCGTGTT	742
cand60_tr5_Sp6_F<br>cand60_tr5_T7_R	Foxj1	ATTTAGGTGACACTATAGATGGTGGGTTCCGATGATTA<br>TAATACGACTCACTATAGGGACAGCAACCACGGAAAAATC	832
cand61_tr5_Sp6_F<br>cand61_tr5_T7_R	Foxj1	ATTTAGGTGACACTATAGAGACTCGGTTTGGCTGAGAA<br>TAATACGACTCACTATAGGGCGCTTCCGATTGATGATTTT	803
cand62_tr5_Sp6_F<br>cand62_tr5_T7_R	Smad4	ATTTAGGTGACACTATAGACAATTTGACAACGCCATGA<br>TAATACGACTCACTATAGGGTACTTTCGGCTTGCTTTCGT	913
cand63_tr5_Sp6_F<br>cand63_tr5_T7_R	H2a	ATTTAGGTGACACTATAGATTCAGGAGCGCATTGGATA<br>TAATACGACTCACTATAGGGGAAGCAGTCTGGTCCGAATC	402
cand64_tr5_Sp6_F<br>cand64_tr5_T7_R	Zmym6	ATTTAGGTGACACTATAGTGTTGGCCAATGCTTCATTA<br>TAATACGACTCACTATAGGGTTGCAGCCAGCTGTTCTCTA	737
cand65_tr5_Sp6_F<br>cand65_tr5_T7_R	Musashi	ATTTAGGTGACACTATAGGATAATGCGAGACCCCGTAA<br>TAATACGACTCACTATAGGGCGTAACAGGAGCAGGGAGAG	721

dsRNA

Name	Gene name	Primer sequences (5'-3')	Product size (bps)
Cand5_dsRNA_T7_F<br>Cand5_dsRNA_T7_R	Ap2	TAATACGACTCACTATAGGGAGGAATTTTGCGAAGAGCAA<br>TAATACGACTCACTATAGGGGCCCGATCCTTGTTGAGTAA	324

Cand6_dsRNA_T7_F<br>Cand6_dsRNA_T7_R	Egr1	TAATACGACTCACTATAGGGGTGACAGCAAATCAACTGTGG<br>TAATACGACTCACTATAGGGGCATGCAGAATAGGAGCAAA	320
Cand10_dsRNA_T7_F<br>Cand10_dsRNA_T7_R	Dr1	TAATACGACTCACTATAGGGTCACAAACTTGCTGGACAGG<br>TAATACGACTCACTATAGGGTGTTGATTATTGGCACCGTC	354
Cand12_dsRNA_T7_F<br>Cand12_dsRNA_T7_R	Smarcb1	TAATACGACTCACTATAGGGTGAGACGGTTCAAATGCTTG<br>TAATACGACTCACTATAGGGACGGAATGAAGAAAATAATCGG	344
Cand14_dsRNA_T7_F<br>Cand14_dsRNA_T7_R	Isl-1	TAATACGACTCACTATAGGGCCGATATCAAAACAACAGAACG<br>TAATACGACTCACTATAGGGTCTCAACCGTTGTCAAATTCC	301
Cand16_dsRNA_T7_F<br>Cand16_dsRNA_T7_R	Lhx2	TAATACGACTCACTATAGGGGGCAACAGACCTTTCAATCC<br>TAATACGACTCACTATAGGGTTGATCATCGGGGAATCAAT	398
Cand17_dsRNA_T7_F<br>Cand17_dsRNA_T7_R	Traf6	TAATACGACTCACTATAGGGAAATCAGAAATGGGGAAATGG<br>TAATACGACTCACTATAGGGGGAAATGCTATTGCTTCGGA	350
