# Supplementary material for: Transcription Factors Active in the Anterior Blastema of Schmidtea mediterranea
Source: Biomolecules. 2021 Nov 28;11(12):1782. doi: 10.3390/biom11121782 (PMC8698962; doi:10.3390/biom11121782)
Supplement: Supplementary file 1 [file biomolecules-11-01782-s001.zip › Table S3.pdf]

Table S3. List of samples used for RNA-seq.

name	description
Bd3-1<br>Bd3-2<br>Bd3-3	anterior blastema manually dissected, day 3 of regeneration
RoBd3-1<br>RoBd3-2<br>RoBd3-3	non regenerating posterior part of the animal from which blastema samples were manually dissected, day 3 of regeneration
Bd6-1<br>Bd6-2<br>Bd6-3	anterior blastema manually dissected, day 6 of regeneration
RoBd6-1<br>RoBd6-2<br>RoBd6-3	non regenerating posterior part of the animal from which blastema samples were manually dissected, day 6 of regeneration
Wt-1<br>Wt-2	Intact, homeostatic control asexual *S. mediterranea*
Irrd3-1<br>Irrd3-2	Irradiated asexual *S. mediterranea*, 3 days after lethal irradiation
Irrd6-1<br>Irrd6-2	Irradiated asexual *S. mediterranea*, 6 days after lethal irradiation
Bd3-1L<br>Bd3-2L	anterior blastema dissected with laser, day 3 of regeneration
RoBd3-1L<br>RoBd3-2L	non regenerating posterior part of the animal from which blastema samples were dissected with laser, day 3 of regeneration
Bd6-1L<br>Bd6-2L	anterior blastema dissected with laser, day 6 of regeneration
RoBd6-1L<br>RoBd6-2L	non regenerating posterior part of the animal from which blastema samples were dissected with laser, day 6 of regeneration
SmB-1<br>SmB-2<br>SmB-3	*SmB*(RNAi) asexual *S. mediterranea*
X1-1<br>X1-2	sorted X1 cells (HR/HB)
x2-1	sorted X2 cells (HR/HB)
x2-2<br>Xin-1<br>Xin-2	sorted Xin cells (HR/HB)
