# Supplementary material for: Transcription Factors Active in the Anterior Blastema of Schmidtea mediterranea
Source: Biomolecules. 2021 Nov 28;11(12):1782. doi: 10.3390/biom11121782 (PMC8698962; doi:10.3390/biom11121782)
Supplement: Supplementary file 1 [file biomolecules-11-01782-s001.zip › Table S4.pdf]

Table S4. Top Blastx hits of the shortlisted candidates.

No.	ID	Gene name	BlastX top hit	Species	Hit ACC	E-Value	Similarity (%)
**1**	tr5_comp12717_c0_seq1	**Musashi**	musashi	*Dugesia japonica*	BAG15902.1	0.0E+00	83
**2**	tr5_comp12153_c0_seq1	**Hsfl**	heat shock factor protein-like isoform X1	*Hydra vulgaris*	XP_012554402.1	1.0E-16	29
**3**	tr5_comp22432_c0_seq1	**Tbx20**	t-box transcription factor tbx20	*S. polychroa*	ADX36149.1	0.0E+00	98
**4**	tr5_comp9443_c0_seq1	**Gata123b**	GATA123b	*S. polychroa*	ADX36140.1	1.0E-126	98
**5**	tr5_comp23253_c0_seq1	**Ap2**	Transcription factor ap-2	*S. mediterranea*	AFJ24713.1	0.0E+00	100
**6**	tr5_comp16049_c0_seq1	**Egr1**	Early growth response protein 1	*S. haematobium*	XP_012792185.1	2.0E-36	71
**7**	tr5_comp16667_c0_seq1	**Zfp**	zinc finger protein	*C. remanei*	EUB65046.1	2.0E-07	36
**8**	tr5_comp12776_c0_seq1	**Myod**	MyoD-like protein	*S. mediterranea*	AAN03696.1	0.0E+00	91
**9**	tr5_comp4620_c0_seq1	**Zfp**	putative zinc finger protein	*S. mansoni*	XP_018654328.1	3.0E-41	33
**10**	tr5_comp9320_c0_seq1	**Dr1**	tata-binding protein-associated phosphoprotein	*S. mansoni*	XP_018646166.1	2.0E-33	50
**11**	tr5_comp16882_c0_seq1	**Lmx1a**	lim homeobox transcription factor 1-alpha-like	*M. yessoensis*	XP_021371147.1	4.0E-34	38
**12**	tr5_comp10797_c0_seq1	**Smarcb1**	swi snf-related matrix-associated actin	*E. granulosus*	CDS21640.1	1.0E-20	28
**13**	tr5_comp4884_c0_seq1	**Ets-1**	ets-1	*S. mediterranea*	AFJ24857.1	9.0E-142	100
**14**	tr5_comp10471_c0_seq1	**Isl-1**	insulin gene enhancer protein isl-1	*S. mediterranea*	AHB51761.1	0.0E+00	87
**15**	tr5_comp15735_c0_seq1	**Nr4a2**	nuclear receptor subfamily 4 group A member 2	*P. vitticeps*	XP_020656813.1	5.0E-47	42
**16**	tr5_comp15800_c0_seq1	**Lhx2**	LIM/homeobox protein Lhx2 isoform X2	*M. zebra*	XP_020501132.1	6.0E-73	42
**17**	tr5_comp9273_c0_seq1	**Traf6**	TNF receptor-associated factor 6	*S. mediterranea*	AFJ24792	1.1E-25	55
**18**	tr5_comp3201_c0_seq1	**Traf5**	TNF receptor-associated factor 5	*Exaiptasia pallida*	KXJ06092	1.7E-41	52
**19**	tr5_comp7406_c0_seq1	**Traf3**	TNF receptor-associated factor 3-like	*Dugesia japonica*	ADF47429	5.1E-22	57
**20**	tr5_comp9449_c0_seq1	**Tbx2**	t-box transcription factor tbx2	*S. polychroa*	ADX36147.1	0.0E+00	98
**21**	tr5_comp9929_c0_seq1	**Smc2**	structural maintenance of chromosomes protein 2	*H. microstoma*	CDS25301.1	0.0E+00	94
**22**	tr5_comp17116_c0_seq1	**Top2**	dna topoisomerase 2	*L. loa*	XP_020306070.1	0.0E+00	49
**23**	tr5_comp18261_c0_seq1	**Fli1**	friend leukemia integration 1 transcription factor isoform 2	*S. mediterranea*	ARX11405.1	0.0E+00	100
**24**	tr5_comp7404_c0_seq1	**Rfc3**	replication factor c subunit 3-like	*A. planci*	XP_022091625.1	6.0E-157	61
**25**	tr5_minimus2_c226	**Tigd1**	tigger transposable element-derived protein 1-like	*C. calcarata*	XP_017891414.1	2.0E-100	60
**26**	tr5_comp4788_c0_seq1	**Etv6**	Transcription factor ETV 6	*S. haematobium*	XP_012796203.1	1.0E-08	58

**27**	tr5_comp4904_c0_seq1	**Tcf15**	transcription factor 15	*S. mediterranea*	AFD29618.1	4.0E-65	100
**28**	tr5_comp5440_c0_seq1	**Taf11**	transcription initiation factor TFIID subunit 11	*S. haematobium*	XP_012795999.1	1.0E-38	63
**29**	tr5_comp5541_c0_seq1	**Elf4**	ets-related transcription factor elf-4-like isoform X2	*M. domestica*	XP_007507217.1	5.0E-14	45
**30**	tr5_comp7051_c0_seq1	**Yeats4**	yeats domain-containing protein 4	*C. sinensis*	GAA38138.2	9.0E-69	50
**31**	tr5_comp7491_c0_seq1	**Pcbp3**	poly(rc)-binding protein 3	*M. yessoensis*	XP_021345121.1	2.0E-60	41
**32**	tr5_comp7710_c0_seq1	**Zgpat**	zinc finger ccch-type with g patch domain-containing protein isoform 2	*O. kisutch*	XP_020314997.1	3.0E-19	31
**33**	tr5_comp7916_c0_seq1	**Rnf11**	ring finger protein 11	*S. japonicum*	CAX69548	4.0E-48	74
**34**	tr5_comp8256_c0_seq1	**Zcchc9**	zinc finger cchc domain-containing protein 9-like	*H. burtoni*	XP_005929100.2	3.0E-25	44
**35**	tr5_comp8854_c0_seq1	**Jmjd2**	jumonji domain containing protein 2	*C. sinensis*	GAA33100.2	3.4E-131	77
**36**	tr5_comp8915_c0_seq1	**H2a**	histone h2a	*E. granulosus*	EUB62025.1	1.0E-50	91
**37**	tr5_comp11360_c0_seq1	**Brd3**	bromodomain-containing protein 3-like	*P. formosa*	XP_007561677.1	8.0E-43	33
**38**	tr5_comp13335_c0_seq1	**Irx3**	iroquois-class homeodomain protein irx-3-like	*O. bimaculoides*	XP_014785002.1	1.0E-41	74
**39**	tr5_comp16074_c0_seq1	**Zmym6**	zinc finger mym-type protein 6	*C. syrichta*	XP_008052733.1	2.0E-72	45
**40**	tr5_comp33150_c0_seq1	**Ep300**	histone acetyltransferase p300 isoform X4	*D. rerio*	XP_009304970.1	8.0E-115	36
**41**	tr5_comp4776_c0_seq1	**Rlm1**	transcription factor Rlm1	*S. cerevisiae*	ONH80884.1	3.0E-08	57
**42**	tr5_comp14042_c0_seq1	**Tufm**	mitochondrial translation elongation factor tu	*B. terrestris*	XP_012168653.1	4.0E-127	53
**43**	tr5_comp10000_c0_seq1	**Dsp1**	high mobility group protein dsp1	*S. haematobium*	XP_012793130.1	8.0E-12	31
**44**	tr5_comp4819_c0_seq1	**Hsf**	heat shock transcription factor	*S. mansoni*	AAC39024.1	6.0E-28	35
**45**	tr5_comp7154_c0_seq1	**Mitfl1**	Microphthalmia associated transcription factor like 1	*S. mediterranea*	AGZ94932.1	0.0E+00	99
**46**	tr5_comp7562_c0_seq1	**Nf-yb**	nuclear transcription factor y subunit beta	*S. mediterranea*	ALX18674.1	1.0E-121	99
**47**	tr5_comp8379_c0_seq1	**Sox2**	Sox b1-2	*S. polychroa*	AIZ72737.1	0.0E+00	93
**48**	tr5_comp8639_c0_seq1	**Prep**	PREP homeodomain-like protein	*S. mediterranea*	ADB54565.1	0.0E+00	99
**49**	tr5_comp9134_c0_seq1	**Nfat5**	nuclear factor of activated t-cells 5-like isoform X2	*B. belcheri*	XP_019629001.1	2.0E-90	57
**50**	tr5_comp12347_c0_seq1	**Prdm1**	pr domain zinc finger protein 1-like	*P. caudatus*	XP_014671079.1	3.0E-58	56
**51**	tr5_comp14240_c1_seq1	**Zfp**	zinc finger protein	*E. granulosus*	EUB65046.1	3.0E-28	76
**52**	tr5_comp14580_c0_seq1	**Six1**	homeobox protein six1	*S. mimosarum*	KFM77431.1	2.0E-114	80
**53**	tr5_comp14844_c0_seq1	**Hr96**	nuclear homone receptor hr96	*E. multilocularis*	CDS43059.1	3.0E-20	55
**54**	tr5_comp14876_c0_seq1	**Fer3l-2**	fer3l-2 protein	*S. mediterranea*	AGZ94910.1	3.0E-103	99

**55**	tr5_comp21339_c0_seq1	**Zica**	zinc finger protein A	*S. mediterranea*	AHW52381.1	0.0E+00	99
**56**	tr5_comp27318_c0_seq1	**Otp**	orthopedia homeobox	*S. polychroa*	AIZ72731.1	3.0E-158	100
**57**	tr5_comp11013_c0_seq1	**Top2a**	dna topoisomerase 2-alpha	*T. adhaerens*	XP_002110327.1	5.0E-105	42
**58**	tr5_comp5898_c0_seq1	**Foxf**	forkhead box F protein	*P. vulgata*	CBI70345.1	3.0E-48	86
**59**	tr5_comp9452_c0_seq1	**Pax2/5/8**	paired box protein pax258	*A. crinita*	ALM30867.1	6.0E-77	84
**60**	tr5_comp9734_c0_seq1	**Foxj1**	forkhead box j1-like protein 4	*S. mediterranea*	AFX62231.1	0.0E+00	99
**61**	tr5_comp11479_c0_seq1	**Foxj1**	forkhead box j1-like protein 2	*S. mediterranea*	AFX62229.1	3.0E-22	42
**62**	tr5_comp23878_c0_seq1	**Smad4**	Mothers Against Decapentaplegic Homolog 4-like	*L. anatina*	XP_013408233.1	2.0E-66	80
**63**	tr5_comp10910_c0_seq1	**H2a**	histone h2a-like, sperm-like isoform X2	*M. persicae*	XP_022165030.1	8.0E-28	64
**64**	tr5_comp20462_c0_seq1	**Zmym6**	zinc finger mym-type protein 6 isoform X1	*S. scrofa*	XP_020951532.1	3.0E-76	44
**65**	tr5_comp7589_c0_seq1	**Musashi**	musashi	*D. japonica*	BAG15904.1	0.0E+00	93
