# Supplementary material for: Transcription Factors Active in the Anterior Blastema of Schmidtea mediterranea
Source: Biomolecules. 2021 Nov 28;11(12):1782. doi: 10.3390/biom11121782 (PMC8698962; doi:10.3390/biom11121782)
Supplement: Supplementary file 1 [file biomolecules-11-01782-s001.zip › Table S5.pdf]

Table S5. GO analysis of the shortlisted, putative TFs and their allocation in the post-hoc groups.

Gene name	DNA-binding protein?	Transcriptional Regulator?	Transcription Factor?	Post-hoc group
**Musashi**	NO	NO	NO	
**Rnf11**	NO	NO	NO	
**Tufm**	NO	NO	NO	
**Smc2**	YES	NO	NO	
**Top2**	YES	NO	NO	
**Pcbp3**	YES	NO	NO	
**H2a**	YES	NO	NO	
**Smarcb1**	YES	YES	NO	TF-G2
**Traf6**	YES	YES	NO	TF-G2
**Traf5**	YES	YES	NO	TF-G2
**Traf3**	YES	YES	NO	TF-G2
**Rfc3**	YES	YES	NO	TF-G1
**Yeats4**	YES	YES	NO	TF-G1
**Zgpat**	YES	YES	NO	TF-G2
**Zcchc9**	YES	YES	NO	TF-G2
**Jmjd2**	YES	YES	NO	TF-G2
**Brd3**	YES	YES	NO	TF-G2
**Ep300**	YES	YES	NO	TF-G2
**Dsp1**	YES	YES	NO	TF-G1
**Hsf**	YES	YES	NO	TF-G2
**Smad4**	YES	YES	NO	TF-G2
**Hsfl**	YES	YES	YES	TF-G1
**Tbx20**	YES	YES	YES	TF-G2
**Gata123b**	YES	YES	YES	TF-G2
**Ap2**	YES	YES	YES	TF-G2
**Egr1**	YES	YES	YES	TF-G2
**Zfp**	YES	YES	YES	TF-G2
**Myod**	YES	YES	YES	TF-G2
**Zfp**	YES	YES	YES	TF-G2
**Dr1**	YES	YES	YES	TF-G1
**Lmx1a**	YES	YES	YES	TF-G2
**Ets-1**	YES	YES	YES	TF-G1
**Isl-1**	YES	YES	YES	TF-G1
**Nr4a2**	YES	YES	YES	TF-G2
**Lhx2**	YES	YES	YES	TF-G2
**Tbx2/3**	YES	YES	YES	TF-G2
**Fli1**	YES	YES	YES	TF-G2

**Tigd1**	YES	YES	YES	TF-G2
**Etv6**	YES	YES	YES	TF-G2
**Tcf15**	YES	YES	YES	TF-G2
**Taf11**	YES	YES	YES	TF-G2
**Elf4**	YES	YES	YES	TF-G2
**Irx3**	YES	YES	YES	TF-G2
**Zmym6**	YES	YES	YES	TF-G2
**Rlm1**	YES	YES	YES	TF-G2
**Mitfl1**	YES	YES	YES	TF-G2
**Nf-yb**	YES	YES	YES	TF-G2
**Sox2**	YES	YES	YES	TF-G2
**Prep**	YES	YES	YES	TF-G1
**Nfat5**	YES	YES	YES	TF-G2
**Prdm1**	YES	YES	YES	TF-G2
**Zfp**	YES	YES	YES	TF-G2
**Six1**	YES	YES	YES	TF-G2
**Hr96**	YES	YES	YES	TF-G2
**Fer3l-2**	YES	YES	YES	TF-G1
**ZicA**	YES	YES	YES	TF-G1
**Otp**	YES	YES	YES	N/C
**Foxf**	YES	YES	YES	TF-G2
**Pax2/5/8**	YES	YES	YES	TF-G2
**Foxj1**	YES	YES	YES	TF-G1
**Foxj1**	YES	YES	YES	TF-G1
**Zmym6**	YES	YES	YES	TF-G2
